# Opening the Gap: Rare Lichens With Rare Cyanobionts – Unexpected Cyanobiont Diversity in Cyanobacterial Lichens of the Order Lichinales

**DOI:** 10.3389/fmicb.2021.728378

**Published:** 2021-10-06

**Authors:** Patrick Jung, Katharina Brust, Matthias Schultz, Burkhard Büdel, Antje Donner, Michael Lakatos

**Affiliations:** ^1^Department of Integrative Biotechnology, University of Applied Sciences Kaiserslautern, Pirmasens, Germany; ^2^Ecology Group, Faculty of Biology, University of Kaiserslautern, Kaiserslautern, Germany; ^3^Institute for Plant Science and Microbiology, Herbarium Hamburgense, University of Hamburg, Hamburg, Germany; ^4^Faculty of Biology, University of Kaiserslautern, Kaiserslautern, Germany

**Keywords:** *Compactococcus*, cyanobionts, Chroococcidiopsidales, *Peltula*, *Komarekiella*, photobionts, *Pseudocyanosarcina*, Lichinales

## Abstract

The last decades of research led to a change in understanding of lichens that are now seen as self-sustaining micro-ecosystems, harboring diverse microbial organisms in tight but yet not fully understood relationships. Among the diverse interdependencies, the relationship between the myco- and photobiont is the most crucial, determining the shape, and ecophysiological properties of the symbiotic consortium. Roughly 10% of lichens associate with cyanobacteria as their primary photobiont, termed cyanolichens. Up to now, the diversity of cyanobionts of bipartite lichens resolved by modern phylogenetic approaches is restricted to the filamentous and heterocytous genera of the order Nostocales. Unicellular photobionts were placed in the orders Chroococcales, Pleurocapsales, and Chroococcidiopsidales. However, especially the phylogeny and taxonomy of the Chroococcidiopsidales genera remained rather unclear. Here we present new data on the identity and phylogeny of photobionts from cyanolichens of the genera *Gonohymenia*, *Lichinella*, *Peccania*, and *Peltula* from a broad geographical range. A polyphasic approach was used, combining morphological and cultivation-depending characteristics (microscopy, staining techniques, life cycle observation, baeocyte motility, and nitrogen fixation test) with phylogenetic analyses of the 16S rRNA and 16S–23S ITS gene region. We found an unexpectedly high cyanobiont diversity in the cyanobacterial lichens of the order Lichinales, including two new genera and seven new species, all of which were not previously perceived as lichen symbionts. As a result, we describe the novel unicellular Chroococcidiopsidales genera *Pseudocyanosarcina* gen. nov. with the species *Pseudocyanosarcina phycocyania* sp. nov. (from *Peltula clavata*, Australia) and *Compactococcus* gen. nov. with the species *Compactococcus sarcinoides* sp. nov. (from *Gonohymenia* sp., Australia) and the new Chroococcidiopsidales species *Aliterella compacta* sp. nov. (from *Peltula clavata*, Australia), *Aliterella gigantea* sp. nov. (from *Peltula capensis*; South Africa), *Sinocapsa ellipsoidea* sp. nov. (from *Peccania cerebriformis*, Austria), as well as the two new Nostocales species *Komarekiella gloeocapsoidea* sp. nov. (from *Gonohymenia* sp., Czechia) and *Komarekiella globosa* sp. nov. (from *Lichinella cribellifera*, Canary Islands, Spain). Our study highlights the role of cyanolichens acting as a key in untangling cyanobacterial taxonomy and diversity. With this study, we hope to stimulate further research on photobionts, especially of rare cyanolichens.

## Introduction

Understanding microbial interactions especially those of symbiotic character — has been and is still a flowering topic across scientific disciplines. In this context, lichens represent an extraordinary example where at least one fungus lives in an intimate association with at least one photosynthetic active alga or cyanobacterium. This complex was redefined as a self-sustaining micro-ecosystem involving many more microbial partners (Farrar, 1976; Hawksworth and Grube, 2020; Grimm et al., 2021). In most cases, eukaryotic green algae, such as members of the green algal family *Trebouxiophyceae*, are frequent partners of lichen mycobionts (chlorolichens), while only 10% of all known lichens have prokaryotic cyanobacteria as their primary partner of choice, called cyanolichens. In terms of their mycobiont–photobiont relationship, cyanolichens can be classified into two functional groups (Rikkinen, 2017): (i) bipartite lichens with one mycobiont and one cyanobacterial photobiont and (ii) tripartite lichens, where one mycobiont associates simultaneously with a green algal and a cyanobacterial photobiont. The latter either form chimeric thalli (photosymbiodemes) or with the cyanobionts restricted to cephalodia, pocket- or gall-like structures in the thallus (Nash, 2001). In tripartite lichens, the cyanobiont is predominantly responsible for nitrogen fixation (Millbank and Kershaw, 1969).

However, Hoffman and Lendemer (2018) found that 89.7% of the molecular studies of lichens published between 2000 and 2016 focused solely on mycobionts, while a minority of studies additionally focused on the photobionts, albeit limited to chlorolichens. Modern DNA sequencing and cultivation techniques allowed, for example, tracking of the photobiont community of *Trebouxia* green algae in chlorolichen populations (Dal Grande et al., 2018) or promoted the identification of specific photobiont lineages (Muggia et al., 2020; Kosecka et al., 2021). In most cases yet, these green algal photobiont lineages reflect geographically restricted “ecotypes,” indicating a cryptic diversity of photobionts, e.g., of the genus *Dictyochloropsis* (Dal Grande et al., 2014). Only in some rare cases were specific green algal photobionts of the *Trebouxia* lineages found to be restricted to a specific lichen species (Sadowska-Deś et al., 2014). These difficulties are corroborated by a low photobiont selectivity in some lichen genera which was detected for the lichen genus *Ramalina*, even within a single habitat that select their photobionts from all phylogenetic groups within the genus *Trebouxia* (Voytsekhovich and Beck, 2016). Insights into the character of the cyanobiont of a referring cyanolichen are rare (Otálora et al., 2010; Magain et al., 2017; Lavoie et al., 2020) and often result solely in molecular data without isolating the photobionts (Dal Forno et al., 2021). Such studies are often restricted to the identification of specific photobiont lineages such as *Nostoc* clades in the case of, e.g., the cyanolichen *Peltigera*, without further taxonomic resolution (Magain et al., 2018). Isolating the cyanobacterial photobionts is crucial because this is the only way to appropriately evaluate, e.g., their taxonomic position. For cyanobacteria, this follows the so-called polyphasic approach (Komárek et al., 2014) that takes into consideration the phylogeny based on the 16S rRNA gene as well as morphological features and ecological aspects such as biogeography. Before applying these standards, other gene regions such as the nucleotide sequences of the cyanobacterial tRNA^Leu^ (UAA) intron indicated that a taxonomic resolution within photobiont lineages within the cyanobacterial genus *Nostoc* appears to be a hard task (Paulsrud et al., 2000). Later, other gene regions such as the protein-coding rbcLX or the 16S rRNA gene were used, coming to the same end of low resolution for this genus (O’Brien et al., 2005). However, up to now, it has not been tested if, for example, the 16S rRNA gene will be sufficient to lead to a greater taxonomic resolution in case of unicellular cyanobacterial photobionts.

In general, the taxonomy of cyanobacteria is perplexed [reviewed in Mareš (2018)], and many questions are left unanswered. Part of the issue is that many studies focus on cyanobacteria from aquatic habitats, while those from terrestrial ecosystems are less intensively studied, although they could close taxonomic gaps at the same time (Osorio-Santos et al., 2014) or broaden the biogeography of established genera (Jung et al., 2020b). Here specific cyanolichens and their photobionts have the potential to widen our view on cyanobacterial taxa involved in lichen symbiosis because the great majority of known symbiotic photobionts belong to filamentous, heterocytous cyanobacterial genera such as *Nostoc* (e.g., in *Collema*, *Lempholemma*, *Leptogium*, *Pannaria*, and *Peltigera*), *Scytonema* (e.g., in *Heppia*), *Stigonema* (e.g., in *Stereocaulon*), *Rhizonema* (e.g., in *Dictyonema* and *Lichinodium*), or *Macrochaete* (e.g., in *Placynthium*). However, there are only very few unicellular cyanobacterial genera involved in bipartite lichen symbiosis, such as *Chroococcidiopsis*, that have, until now, never been confirmed within the backbone of molecular techniques—for example, photobionts of bipartite lichens within *Lichinaceae* such as *Anema*, *Peccania*, *Psorotichia*, and *Peltula* were assigned to *Chroococcidiopsis* based on morphology (Büdel and Henssen, 1983) and later partly confirmed by 16S rRNA gene studies (Fewer et al., 2002). *Chroococcidiopsis lichenoides* is the only fully described species within the whole order Chroococcidiopsidales which is fully described after modern criteria as a photobiont of an undescribed tripartite lichen (Villanueva et al., 2018).

The genus *Chroococcidiopis* is the basis of the order Chroococcidiopsidales whose members often have characteristics of true extremophiles living in hot and cold deserts. Among them are genera such as *Chroococcidiopsis*, *Gloeocapsopsis*, and *Aliterella* occurring in deserts (Büdel, 1999; Jung et al., 2019, 2021) and being extremely desiccation tolerant (Azua-Bustos et al., 2014). They even have been proposed as model organisms for the colonization of Mars (Billi et al., 2019; de Vera et al., 2019; Mosca et al., 2019). Besides the supplied photosynthesis products, it is still a debate if these chroococcidiopsidalean strains have the additional benefit of fixing atmospheric nitrogen for the mycobiont because they possess genes for nitrogen fixation but, so far, did not grow on nitrogen-depleted media (Billi and Caiola, 1996; Ramos et al., 2010). Despite this negative growth success, nitrogen fixation of free-living (from gypsum rock) and lichenized (cyanolichen *Thyrea* spp.) populations of *Chroococcidiopsis* spp. was confirmed in the field and in the laboratory (Boison et al., 2004; Crittenden et al., 2007).

However, the taxonomic and phylogenetic positions of *Chroococcidiopsis* spp. were recently questioned (Wang et al., 2019; Jung et al., 2021) due to emerging evidence that the order appears to be perplexed. The underlying study would like to further close the knowledge gap of unicellular cyanobionts by elucidating isolated photobionts of three lichen specimens from the genera *Peltula* (Australia and South Africa), two of *Gonohymenia* (Canary Islands, Spain and Czechia), one of *Peccania* (Austria), and one from *Lichinella* (Canary Islands, Spain), following the polyphasic approach as well as additional analyses. The photobionts of all lichens were isolated and analyzed based on their 16S–23S rRNA gene sequences, and details on morphology were obtained from multiple microscopy techniques. Additionally, the strains were tested for motility to differentiate them from pleurocapsalean taxa, which share some morphological features but differ by their motile baeocytes. For testing dinitrogen fixation capabilities, the isolates were grown on a nitrogen-free medium. Taking all of these parameters together, we intend to provide facts to resolve the degree of phylogenetic relationship of the cyanobionts following the rules and requirements of the International Code of Nomenclature for algae, fungi, and plants (Turland et al., 2018).

## Materials and Methods

### Origin of Lichens and Isolation of Photobionts

All lichens were collected from rocks throughout various field trips by Aino Henssen and Burkhard Büdel. In detail, the lichen specimens of *Lichinella cribellifera* were collected on the Canary Islands, Spain, *Gonohymenia* sp. in the Czechia and Australia, *Peltula clavata* in Australia, *P*. *capensis* in the South Africa, and *Peccania cerebriformis* in Austria (Table 1). Collaboration partners, the government, or ranger associations of the specific regions and countries were involved in the sampling procedure, and restrictions at that time were followed, including sampling permits, shipping, and tax. Furthermore, the strains and lichens are compliant because they were collected well before the effective date of the Convention of Biological Diversity and the Nagoya Protocol. The respective documents were given to the public herbaria and culture collections where the lichens and photobionts were deposited.

**TABLE 1:** Overview of the new cyanobionts, the respective lichen and location of collection, and the final German Collection for Microorganisms and Cell Cultures (DSMZ) strain number as well as NCBI GenBank accession numbers and holotype deposition.

Lichen	Origin	Photobiont	DSMZ strain number	Herbarium Hamburgense holotype number	NCBI GenBank accession number
*Gonohymenia* sp.	1981, Czechia	*Komarekiella gloeocapsoidea*	112644	HBG-025111	MZ160908
*Gonohymenia* sp.	1987, Australia	*Compactococcus sarcinoides*	112643	HBG-025112	MZ160914
*Lichinella cribellifera*	1989, Canary Islands, Fuerteventura, Spain	*Komarekiella globosa*	112645	HBG-025113	MZ160911
*Peltula clavata*	1987, Australia, Queensland, seepage rock 2 m a.s.l.	*Pseudocyanosarcina phycocyania*	112640	HBG-025114	MZ160910
*Peltula clavata*	1987, Australia, Queensland, seepage rock 2 m above a river	*Aliterella compacta*	112641	HBG-025115	MZ160912
*Peltula capensis*	1994, South Africa, Limpopo Province, Vhembe Nature Reserve, temporary submerged sandstone, in a seasonal flooded riverbed, 600 m a.s.l.	*Aliterella gigantea*	112646	HBG-025116	MZ160909
*Peccania cerebriformis*	1979, Austria, near Graz, on rock	*Sinocapsa ellipsoidea*	112642	HBG-025117	MZ160913

In order to isolate the photobionts, the lichens were removed from their substrate with tweezers. Afterward, the lichen fragments were washed in sterile water in order to remove adhering contaminants and rock fragments. Subsequently, the fragments were submerged in 4% hydrogen peroxide solution to eliminate algae as well as heterotrophic contaminants from the surface of the lichen thalli. The cleaned parts of the lichens were collected in sterile 1.5-ml tubes filled with 500 μl BG 11 medium [prepared after Stanier et al. (1971)], and the photobiont cells were deliberately crushed with potter sticks. Alternatively, the photobionts were isolated from thin sections of the lichen specimen prepared with a freezing microtome. The cyanobionts were picked with sterile tweezers and transferred to liquid BG 11 medium. The resulting solution was transferred on BG 11 agar plates and incubated in a culture room at 17°C at a light intensity of 30 μmol m^–2^s^–1^ and a light/dark cycle of 18:6 h for several weeks.

The colonies on the agar plates were checked weekly until cyanobacterial colonies that grew out of lichen thallus fragments could be picked with a sterile needle and transferred to fresh agar plates containing BG 11 medium. Taking only colonies that grew in contact with lichen fragments indicated that the isolates were most likely the photobionts of the lichens but, at the same time, hampered the establishment of algal cultures free of heterotrophic contamination because fungal material from the environmental sample was always attached to the photobionts. A serial transfer of the colonies to fresh agar plates over the course of several months thinned out the contamination and finally provided fungus-free cultures. In some cases where fungal contamination was not eliminated, the contaminated cyanobacterial colonies were transferred to BG 11 plates containing 0.2% nystatin. Finally, the purified photobiont isolates were kept in liquid BG 11 under the conditions described.

### Motility and Nitrogen Fixation Tests

The motility of baeocytes acts as a discrimination feature between unicellular pleurocapsalean and chroococcidiopsidalean taxa (Waterbury and Stanier, 1978). Thus, a motility test was applied to all isolates by transferring small proportions of the cultures on one side of agar plates with BG 11 medium. This side of the plate was fully covered with a black adhesive tape and placed in a culture cabinet (CLF Plantclimatics, Percival), as described above, in a way that the uncovered side was directed orthogonally to the light source. Over the course of 3 months, it was regularly checked if the cells are motile at any stage of their developmental cycles and move toward the light.

The nitrogen fixing abilities of the strains were also checked when they were transferred to agar plates (Difco Bacto Agar, Becton Dickinson) with nitrogen-free BG 11 medium and kept under the same cultivation conditions as described above for at least 4 months. During this time, they were regularly inspected by light microscopy and transferred to fresh BG 11 agar plates with nitrogen to check for their recovery.

### Morphological Characterization

To ensure collecting a comparable maximum of the morphological features of the cyanobiont isolates, their life cycles were studied on BG 11 agar plates as well as in liquid BG 11 medium by light microscopy. In addition, all strains were checked for their ability to fix nitrogen from the air by cultivating them separately on BG 11 agar plates without nitrogen for about 4 months. All agar plates were sealed with parafilm to prevent the desiccation of the agar and the cells.

Morphological characteristics were evaluated with a Panthera KU trinocular light microscope (Motic) coupled with a MicroLive Multi Format camera and the software MicroLive (v4.0). About 200 cells were measured, and a mean size range is given, reflecting the variability of irregular cell sizes. In addition to bright-field images, samples were analyzed under simple polarized light, autofluorescence, Indian ink staining, and ACN staining. The latter is a 20:1:1 mix of Astra Blue, chrysoidin, and Neufuchsin (0.1 g Astra Blue in 79.5 ml H_2_O and 2.5 ml acetic acid, 0.1 g chrysoidin in 100 ml H_2_O, and 0.1 g Neufuchsin in 100 ml H_2_O) that allows a differentiation of structures according to color due to the binding characteristics of the substances. Acid mucopolysaccharides are stained blue by Astra Blue, while cellulose or lignin is stained red by Neufuchsin and hydrophobic substances such as cutin yellow.

For some strains, low-temperature scanning electron microscopy (SEM) was applied (Zeiss, Oberkochen, Germany) as described in Jung et al. (2020a).

For transmission electron microscopy studies, recently collected material was soaked in tap water for roughly 6 h and subsequently fixed in non-buffered aqueous 1% KMnO_4_ solution for 1 h. After fixation, the samples were dehydrated in a series of increasing ethanol–water solutions, up to 100% ethanol. Samples were then embedded in epoxy resin (Spurr, 1969) and cut with a LKB ultra-microtome (Medical Instrumentation Inc., Florida, United States) using a diamond knife. Ultrathin sections were contrasted using uranyl acetate and lead citrate (Watson, 1958; Reynolds, 1963). For examination and documentation, a Phillips 301 G electron microscope (Amsterdam, Netherlands) was used.

### Molecular Characterization

Roughly 20 mg of culture material was scraped off from agar plates and collected in 1.5-ml tubes with 300 μl SoluLyse, a lysis buffer for the protein extraction of bacteria (Amsbio, England). The samples were crushed manually with potter sticks and incubated overnight at room temperature on a shaking plate at 110 rpm (Unimax 1010; Heidolph Instruments GmbH & Co.KG, Kehlheim, Germany). Afterwards, 300 μl of buffer B (1.4 M NaCl, 20 mM Na_2_-EDTA, 100 mM Tris–HCl, pH 8.6) was added and vortexed for a few seconds. DNA was purified by adding 500 μl chloroform/isoamyl alcohol (24:1), shaking for 5 min, and centrifuging for 5 min at 11,000 *g*. The resulting upper phase was transferred to a new 1.5-ml tube, and 500 μl of phenol/chlorophorm (1:1) was added. After shaking and centrifuging, the chloroform/isoamyl step was repeated, and the supernatant was again collected, to which 1/10 (v/v) sodium acetate and 2/3 (v/v) of isopropanol were added in order to precipitate the extracted genomic DNA in the freezer at −20°C overnight. Finally, DNA pellet was obtained by spinning the DNA down at 14,000 *g* for 20 min, which was washed with 500 ml pre-chilled ethanol (70%) and resuspended in 100 μl ddH_2_O.

The 16S–23S ITS gene region was amplified by PCR in a 50-μl reaction using the primers Wil1 and Wil18 (Wilmotte et al., 1993) and ready-to-go PCR mini beads (GE Healthcare) as described in Jung et al. (2021). The quality of the PCR products was checked by means of agarose gel electrophoresis using 1% (w/v) agarose and subsequently purified with NucleSpin Gel and PCR Clean-up Kit (Macherey-Nagel GmbH & Co. KG, Düren, Germany) following the DNA and PCR cleanup protocol. Purified PCR products were sent for Sanger sequencing to Genewiz (Germany GmbH, Leipzig, Germany) using the primers Wil1, Wil4, Wil5, Wil10, Wil11, Wil16, and Wil18. For some cases where the amplification was not successful, the alternative primers SSU-4-forw and ptLSU C-D-rev were used in a 25-μl reaction as described by Marin et al. (2005). Here PCR products were sent for Sanger sequencing with the primers SSU-4-for, Wil 6, Wil 12, Wil 14, Wil 5, Wil 9, Wil 16, and ptLSU C-D-rev.

The generated sequences were assembled with Geneious Prime (v2021.0.1) software package (Biomatters Limited, New Zealand). The sequences were submitted to the National Center for Biotechnology Information (NCBI) GenBank, as stated in the species description, with accession number MZ160908-14 (Table 1). The 16S rRNA gene sequences were compared with those of known species using the NCBI GenBank BLAST tool, and the percentage of identity with other 16S rRNA gene sequences is given in the species descriptions.

The assembled 16S rRNA gene sequences obtained from the isolates and related sequences of cyanobacterial strains cited from GenBank were used for phylogenetic analyses, including *Gloeobacter violacaeus* as outgroup for the 16S rRNA gene alignment, applying the Muscle algorithm in Mega X (Kumar et al., 2018). Three separate alignments were prepared: one to give a state-of-the-art overview about all orders, a second one depicting the most recent in-depth phylogeny of the Nostocales, and a third one simulating the proposed monophyly of the order Chroococcidiopsidales (Komárek et al., 2014) by excluding complete 16S rRNA gene sequences of specific Oscillatoriales that split some genera within the Chroococcidiopsidales. This specific split was previously detected by Wang et al. (2019) and Jung et al. (2021) and is induced by the 16S rRNA gene sequences of *Cephalothrix lacustris*, *Aerosakkonema funiforme*, and *Microseira wollei* as well as the Oscillatoriales “core group” including members of the genera *Wilmottia*, *Microcoleus*, and related taxa.

Finally, 179 nucleotide sequences were used for the phylogenetic comparison of the unicellular isolates, including 1,438 bp of the 16S rRNA gene with representatives from all orders, 71 sequences comprising 1,505 bp giving details about the phylogeny of the nostocalean genera, and 92 sequences with 1,438 bp of the chroococcidiopsidalean genera only. Single ambiguous base pairs within otherwise highly conserved regions within the alignments were adjusted or removed manually, allowing smaller final blocks and gap positions within the final blocks. The evolutionary model that was best suited to the database used was selected on the basis of the lowest Akaike information criterion value and calculated in Mega X for all trees. The phylogenetic tree was finally constructed with Mega X using the evolutionary model RGT + G + I of nucleotide substitutions. The maximum likelihood method (ML) with 1,000 bootstrap replications was calculated with Mega X and Bayesian interference (BI) phylogenetic analyses, with two runs of eight Markov chains executed for one million generations with default parameters with Mr. Bayes 3.2.1 (Ronquist and Huelsenbeck, 2003) for all trees. Each analysis reached stationarity (average standard deviation of split frequencies between runs < 0.01) before the end of the run.

Models of the secondary structure of the 16S–23S ITS gene region of all isolates were built in comparison to phylogenetic or morphologically related genera such as *Chroococcidiopsis thermalis* PCC 7203 according to the models proposed in Zhang et al. (2018). Helices were folded with default setting using the online software RNAstructure Web Server (Reuter and Mathews, 2010).

### Holotype Preparation

New genera and species were described following the rules and requirements of the International Code of Nomenclature for algae, fungi, and plants (Turland et al., 2018). For the preservation of the type strains, young, 3-week-old cultures were transferred into 5-ml glass bottles with a 4% (v/v) formaldehyde–water mixture. The preserved material is deposited at Herbarium Hamburgense, Hamburg, Germany (Table 1), while the living culture material is given to the German Collection for Microorganisms and Cell Cultures (DSMZ), Braunschweig, Germany (DSM 112640–112646; Table 1).

## Results

An evaluation of the isolated cyanobiont strains in the context of the polyphasic approach (Komárek et al., 2014) and additional ecologically based analyses indicated the establishment of the new species *Aliterella gigantea*, *A. compacta*, *Sinocapsa ellipsoidea*, *Komarekiella gloeocapsoidea*, and *K. globosa* but also led to the description of the two novel genera *Compactococcus* with *C. sarcinoides* and *Pseudocyanosarcina* with *Pseudocyanosarcina phycocyania*. In the following sections, their phylogenetic position, morphology, and the resulting taxonomic treatment will be depicted and summarized in Table 1.

### Phylogeny Based on the 16S rRNA Gene

**FIGURE 1:**
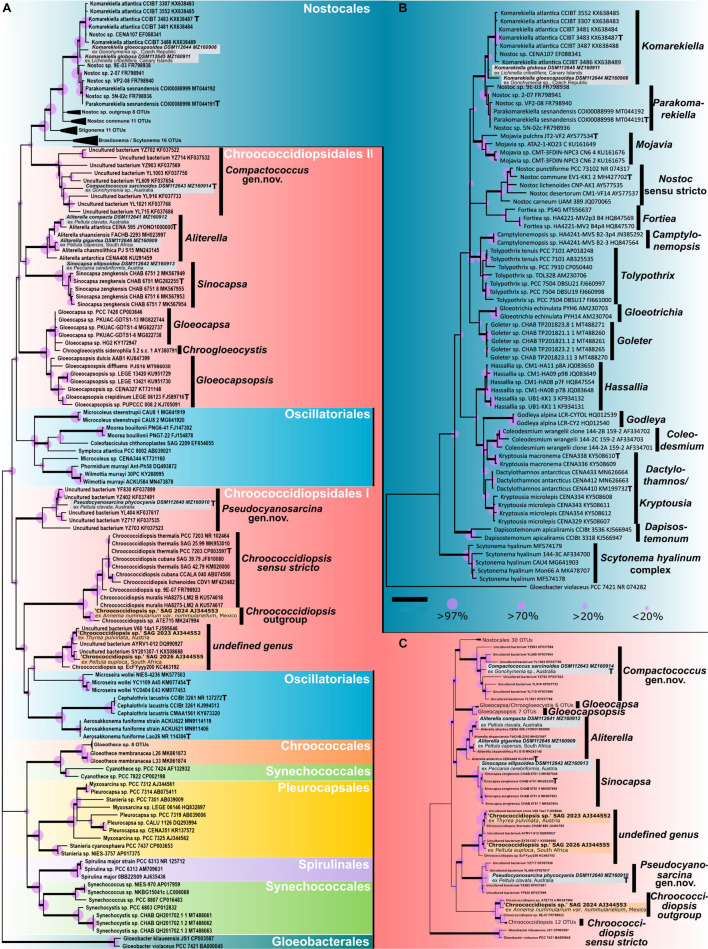
Phylogenetic maximum likelihood (ML) trees based on the 16S rRNA gene region of the isolated photobionts. **(A)** ML phylogenetic tree comprising 105 sequences (1,438 bp) of representatives from all cyanobacterial orders rooted to *Gloeobacter*, giving an overview of the almost complete taxonomy of the phylum. **(B)** ML phylogenetic tree comprising 71 sequences (1,505 bp) of main nostocalean representatives rooted to *Gloeobacter*, giving a detailed overview of the nostocalean taxonomy. **(C)** ML phylogenetic tree comprising 92 sequences (1,438 bp) of all chroococcidiopsidalean representatives with Nostocales as outgroup rooted to *Gloeobacter*. Here the 16S rRNA gene sequences of Oscillatoriales that split Chroococcidiopsidales in **(A)** were excluded to simulate a monophyly of the order Chroococcidiopsidales as previously supposed. The three sequences in orange (*Chroococcidiopsis* sp. SAG 2023 from *Thyrea pulvinata*, SAG 2024 from *Anema nummularium var. nummulariellum*, and SAG 2026 from *Peltula euploca*) are from additional photobionts that were not investigated during this study and possibly indicate two additional undescribed chroococcidalean genera. The type strains of the genera are indicated with a bold T. For each sequence, the strain number as well as the NCBI GenBank accession number is displayed. Since the resulting Bayesian and ML phylogenetic trees mostly showed the same topology, a single tree with both Bayesian and ML bootstrap values is shown. Supports at the nodes (Bayesian interference (BI)/ML) represent posterior probabilities, and bootstrap values indicated as purple circles of different sizes refer to percent intervals. Nodes greater than 85% statistical support from both; ML and BI are highlighted in bold. The scale bar specifies 0.05 expected changes per site for each of the phylogenetic trees.

In general, Figure 1 depicts the most recent cyanobacterial phylogeny based on 16S rRNA gene sequences comparable to those presented by Wang et al. (2019) or Jung et al. (2021). Here the most recent and thoroughly described type strains of the genera (indicated with a T in Figure 1) that are important for a comparison with 16S rRNA gene sequences from our strains were included, such as *Chroococcidiopsis thermalis* PCC 7203, *Gloeocapsopsis crepidinum* LEGE 06123 (Jung et al., 2021), *Aliterella atlantica* CENA 595 (Rigonato et al., 2016), *Komarekiella atlantica* CCIBt 3483 (Hentschke et al., 2017), *Sinocapsa zengkensis* CHAB6571 (Wang et al., 2019), or *Parakomarekiella sesnandensis* coi00088998 (Soares et al., 2020).

The phylogeny shown in Figure 1A indicates that the 16S rRNA gene sequences of the two strains DSM 112645 and DSM 112644 clustered in the Nostocales at a mid-position between the clades describing the genera *Komarekiella* and *Parakomarekiella*. The full resolution of the nostocalean phylogeny shown in Figure 1B shows that they, together with *K. atlantica* and *P. sesnandensis*, formed a bigger well-supported single clade in close relation to the genus *Mojavia*. Both strains were highly similar to *P. sesnandensis* (98%) and to *K. atlantica* (98–99%).

Considering the almost complete taxonomic range comprising members of all orders of cyanobacteria, the strains DSM 112640–43 and DSM 112646 fell into the unicellular order Chroococcidiopisidales within several well-supported clades (Figure 1A). Here the genus *Aliterella* clusters together with *Sinocapsa*, and *Gloeocapsopsis* forms a well-supported bigger clade with *Gloeocapsa* and *Chroogloeocystis*, while the newly established genus *Compactococcus* is well supported at the base of the Nostocales. Together these genera form the herein termed group “Chroococcidiopsidales II” that is split apart by Oscillatoriales from the genus *Chroococcidiopsis*, the newly established genus *Pseudocyanosarcina*, and two possibly new genera made of additional photobionts that are herein named “Chroococcidiopsidales I.” In order to simulate the until now proposed monophyly of the order Chroococcidiopsidales (Komárek et al., 2014), a second phylogenetic tree was prepared (Figure 1C). There the Oscillatoriales was excluded from the analysis, with the result that the positioning of the chroococcidiopsidalean genera remained identical to the full phylogeny depicted in Figure 1A but with a slightly better statistical support. However, the gap where the Oscillatoriales inserted the split between Chroococcidiopsidales I and II in the full phylogeny (Figure 1A) was also clearly visible in Figure 1C.

In detail, strains DSM 112646 and DSM 112641 clustered within the chroococcidiopsidalean genus *Aliterella* (Figures 1A,C), and their 16S rRNA gene shared between 96.91 and 99.17% similarities to other members of this genus, while DSM 112642 clustered together with the related genus *Sinocapsa* (*S. zengkensis*) (Figures 1A,C), sharing 98% identity based on the 16S rRNA gene. The two strains DSM 112640 and DSM 112643 did not fit into any existing cluster describing a distinct genus within the Chroococcidiopsidales but formed two well-supported and separate clusters with highly similar 16S rRNA sequences of uncultured cyanobacteria from the Karst area of the Stone Forest in Yunnan, China (Ren et al., 2018), at different positions within the tree (Figures 1A,C). The addition of 16S rRNA gene sequences from photobionts available *via* public culture collections resulted in a yet undescribed but well-supported sister taxon to *Chroococcidiopsis* and in a second undefined, well-supported cluster together with 16S rRNA gene of uncultured (cyano)bacteria (Figures 1A,C; orange).

### Strain Morphology, Life Cycle, Baeocyte Motility, and Nitrogen Fixation

**FIGURE 2:**
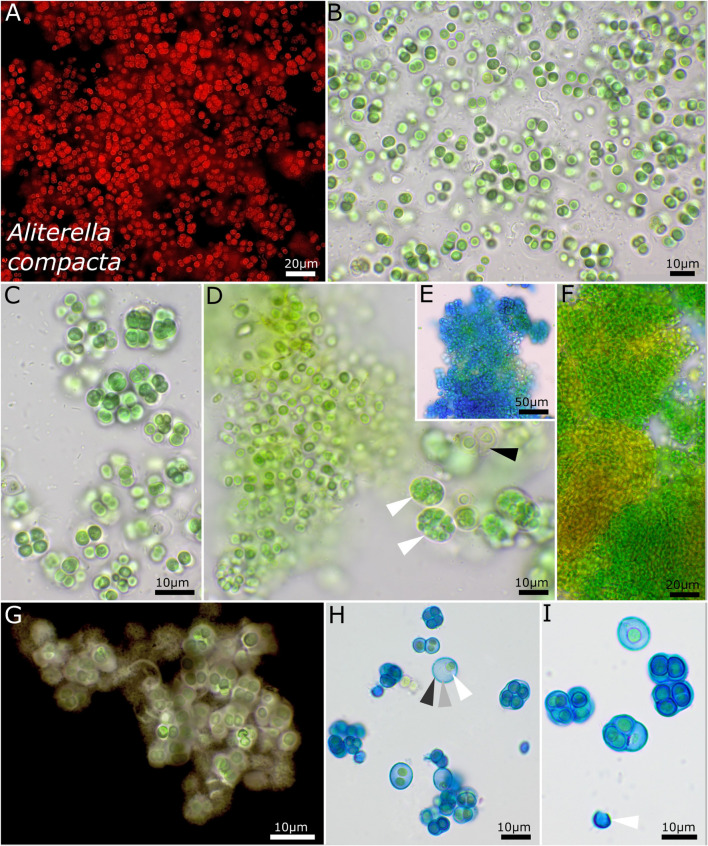
Micrographs showing *Aliterella compacta*. **(A)** Fluorescence microscopy and **(B–I)** light microscopy. **(A–C)** Rounded single cells embedded in rounded, limited sheaths. **(D)** White arrows indicate compact groups of young, pressed cells encapsulated in firm sheaths (= baeocytes); black arrow points toward wide sheaths of older cells. **(E)** Stained with ACN solution colorizing firm and non-diffluent sheath material of colonies. **(F)** Single cells arranged in dense colonies, with older parts turning yellow. **(G)** Indian ink staining of colony indicating firm, non-diffluent sheath matrix. **(H,I)** ACN staining showing limited and lamellate sheath material with three layers as marked with arrows in **(H)** and an empty, capsule-like sheath in **(I)**.

**FIGURE 3:**
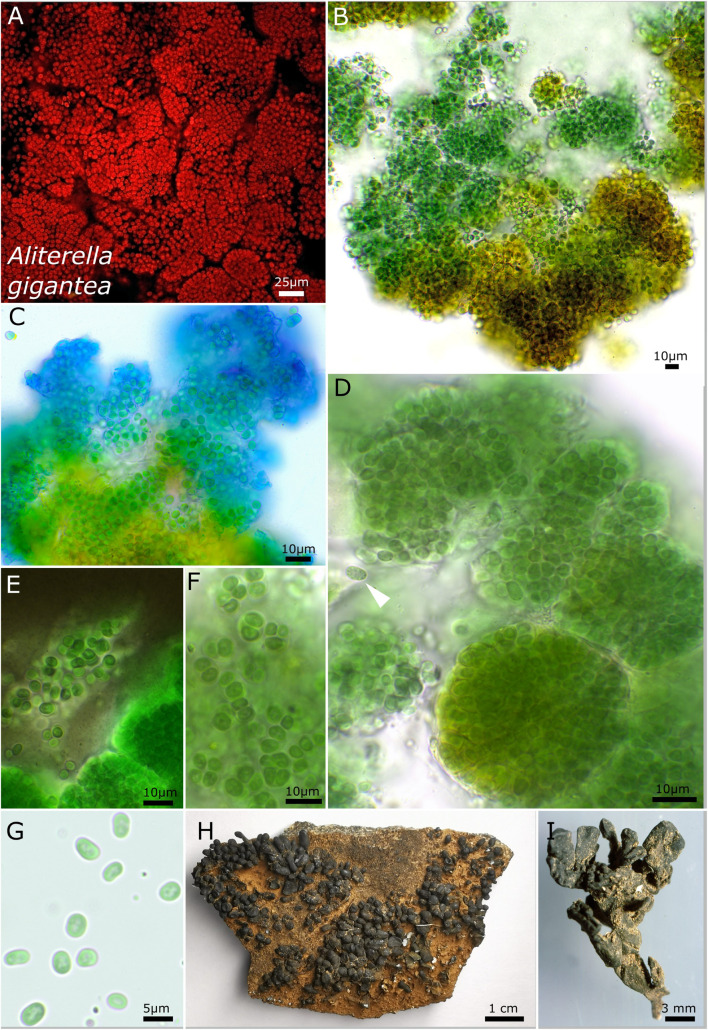
Micrographs showing *Aliterella gigantea*. **(A)** Autofluorescence image, **(B–G)** light microscopy, and **(H,I)** photographs of the lichen. **(B)** Yellow color of the peripheral parts of the colony. **(C)** ACN staining indicating the firm sheath matrix of larger colonies. **(D)** Package-like colonies made of hundreds of single cells enclosed in firm, hyaline sheaths; the white arrow points toward unsheathed single cell with fine granules at the periphery of the cell. **(E)** Indian ink staining highlighting the firm sheath of cell packages and unsheathed single cells. **(F)** Small groups of a few cells with parietal thylakoid membrane. **(G)** Unsheathed single cells with thylakoid-free lumen in the center of the cells. **(H)** Overview photograph of *Peltula clavata*. **(I)** Close-up photograph of lichen thallus.

The unicellular strain DSM 112641 is characterized by cell packages made of a few dozen cells tightly pressed against each other with a firm and limited sheath that expands during aging and becomes lamellate comprising enlarged cells (Figure 2). In contrast, strain DSM 112646 is differentiated by up to 6 μm large, ellipsoidal cells that are not tightly pressed against each other in larger colonies (Figure 3).

**FIGURE 4:**
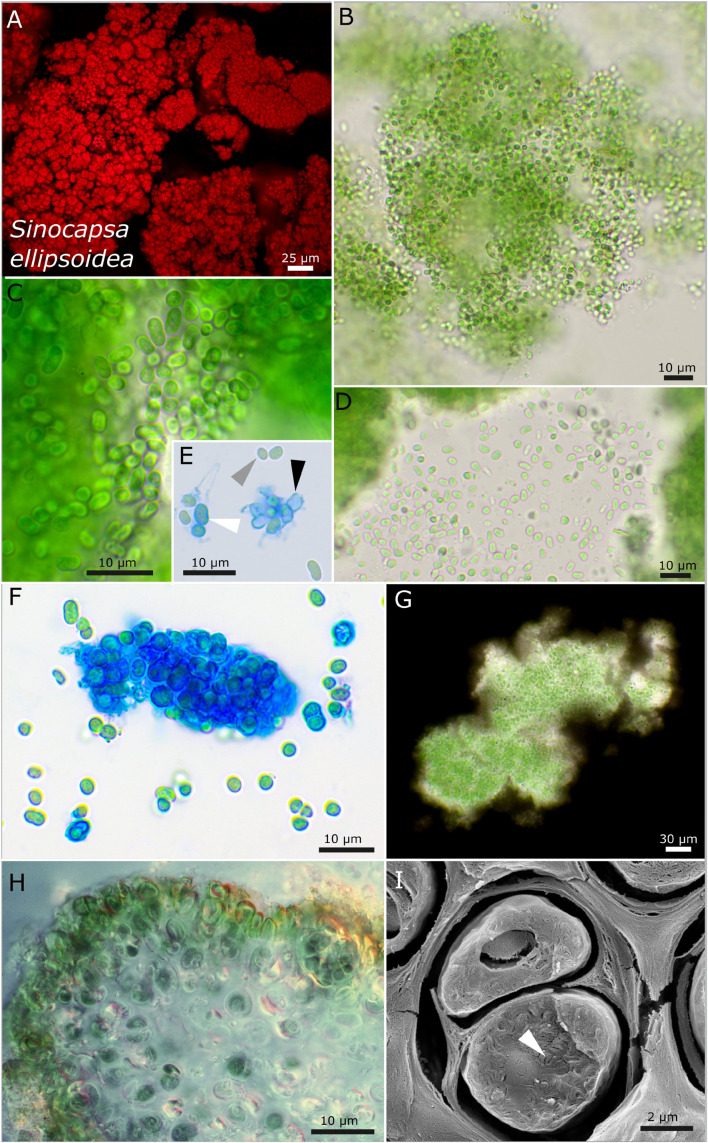
Micrographs showing *Sinocapsa ellipsoidea*. **(A)** Autofluorescence image, **(B–H)** light microscopy, and **(I)** low-temperature scanning electron microscopy (SEM). **(B)** Overview image showing the diffluent character of larger colonies. **(C,D)** Released, unsheathed, ellipsoidal single cells. **(E,F)** ACN staining highlighting the capsule-like, firm sheath material [white arrow in **(E)**], unsheathed cells [gray arrow in **(E)**], and empty capsules [black arrow in **(E)**]. **(G)** Indian ink staining visualizing the sheath matrix of larger colonies. **(H)** Cross-section through a thallus piece of *Peccania cerebriformis* showing the rounded cyanobacteria. **(I)** SEM image of two cut cells with their coiled thylakoid membranes (white arrow).

Strain DSM 112642 has ellipsoid cells that are slightly bent with a tight, capsule-like sheath and unsheathed cells after release from colonies (Figure 4). The photobiont cells are embedded in a mucilaginous matrix in the lichen, and the cells possess coiled thylakoid membranes that do not fill out the whole cell (Figure 4I).

**FIGURE 5:**
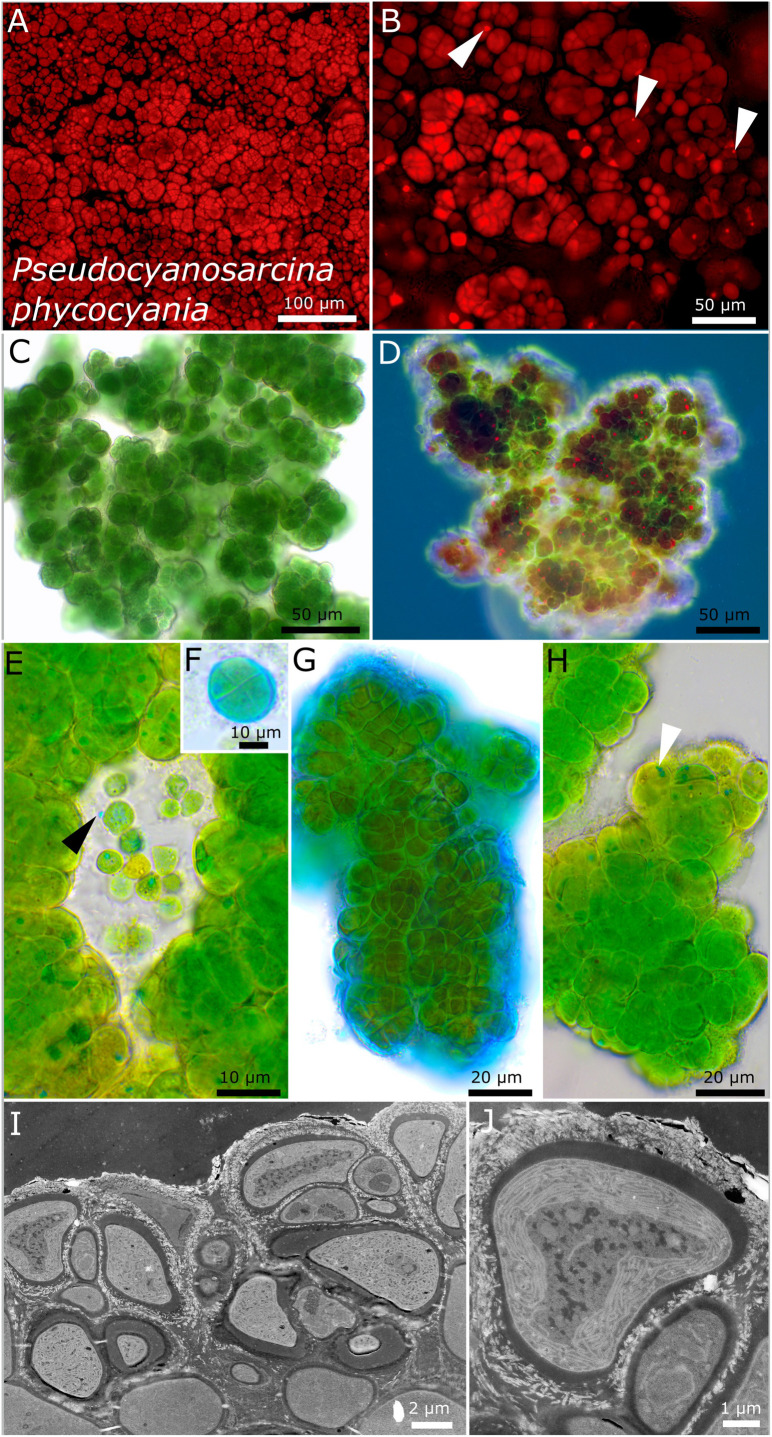
Micrographs showing *Pseudocyanosarcina phycocyania*. **(A,B)** Fluorescence images showing phycocyanin granules [white arrows in **(B)**]. **(C–H)** Light microscopy and **(I,J)** transmission electron microscopy (TEM) of lichen cross-sections. **(C)** Overview showing the rounded structure of the colonies. **(D)** Larger colony under simple polarized light with phycocyanin granules in red. **(E)** Close-up of released, unsheathed single cells with blue phycocyanin granules that can be excreted outside of the cells (black arrow). **(F,G)** ACN staining showing a round tetrad of four cells and the firm, non-lamellate, limited sheath. **(H)** Colony made of dense cells, tightly pressed against each other and forming rounded packages comprising a few cells with blue phycocyanin granules (white arrow). **(I,J)** TEM images showing the cell of the photobiont arranged in the lichen thallus and the lamellar, parietal position of the thylakoid membranes within the cells.

Compared to all investigated strains, strain DSM 112640 has large cells (up to 8 μm) with a morphology comparable to that of the pleurocapsalean genus *Cyanosarcina* (Figure 5). As a frequent character, blue phycocyanin granules inside and outside of the cells were observed (Figure 5E). The photobiont cells are slightly smaller in the lichen thallus with a parietal, lamellar arrangement of the thylakoid membranes that do not fill out the whole cell (Figures 5I,J).

**FIGURE 6:**
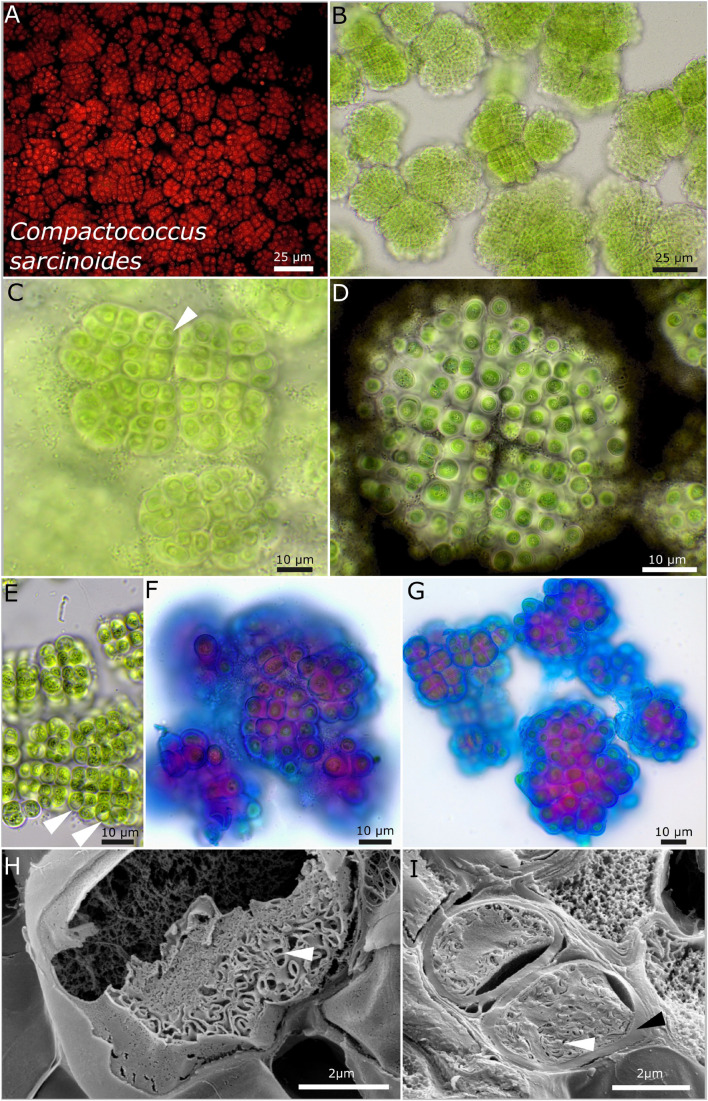
Micrographs of *Compactococcus sarcinoides*. **(A)** Autofluorescence, **(B–G)** light microscopy, and **(H,I)** low-temperature scanning electron microscopy (SEM). **(B)** Overview of colonies arranged in equal packages made of rows of cells. **(C)** Close-up of small colonies with older cells that initially form vacuole-like structures (white arrow). **(D)** Indian ink highlighting the limited and lamellate character of the sheaths surrounding each single cell. Note the equal arrangement of the cells in rows and the division cross-dividing the colony in four quadrants. **(E)** Older colonies with vacuole-like structures bloating the cells (white arrow) and fine, crystalline granules in the center of the cells. **(F,G)** ACN staining coloring acid polysaccharides in blue that form the periphery of each sheath and inner sheath structure stained in purple due to a changing composition of the polysaccharides. **(H,I)** SEM images showing cut cells with thick capsule-like cell walls (black arrow) and the thylakoid membranes (white arrows).

Rounded to slightly rectangular patterns of the colonies are characteristic features of strain DSM 112643. This is because of timed binary fission of the cells (Figure 6). By forming vacuole-like structures, older cells got a bloated habitus (Figure 6E). ACN staining unveiled a stratification of the extracellular polymeric substances with acidic polysaccharides at the periphery of the sheaths stained in blue (Figures 5F,G). The thylakoid membranes of the cells are coiled to slightly tubular and placed at the cell periphery (Figures 5H,I).

**FIGURE 7:**
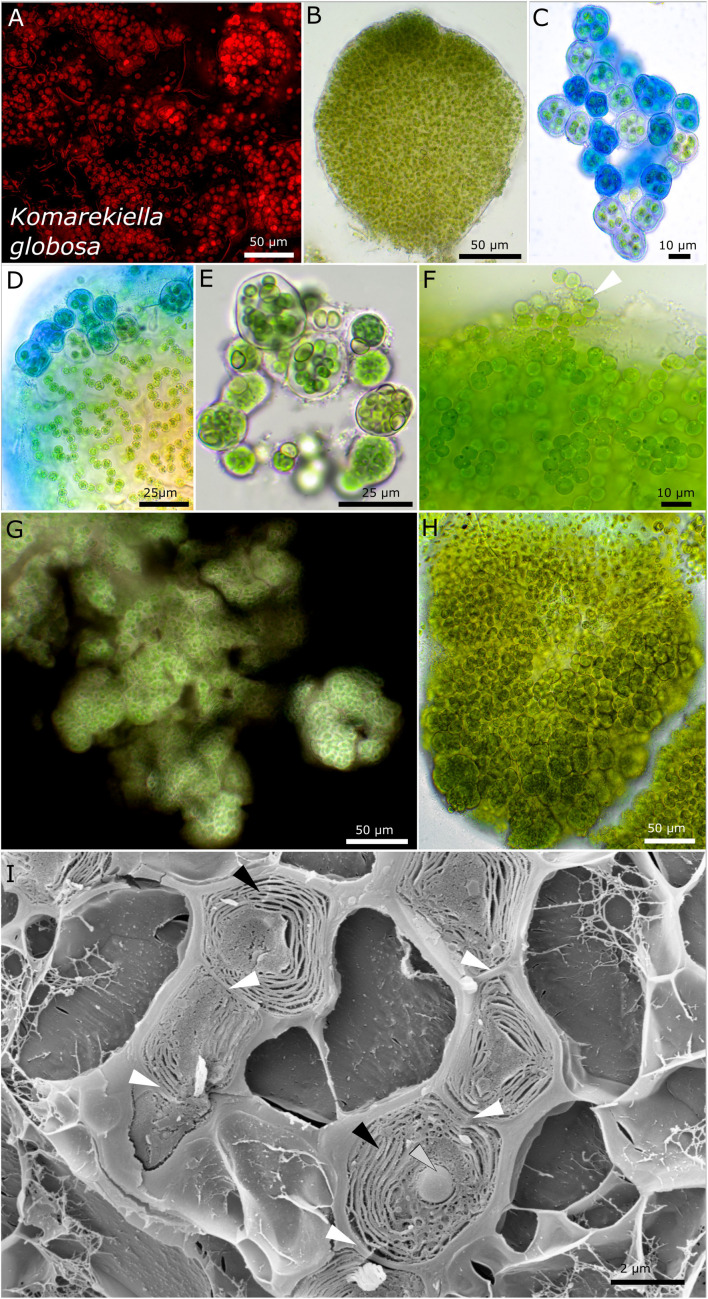
Micrographs of *Komarekiella globosa*. **(A)** Autofluorescence, **(B–H)** light microscopy, and **(I)** low-temperature scanning electron microscopy (SEM). **(B)** Cross-section through a globose, macroscopic colony. **(C)** ACN staining of young colonies showing the limited, hyaline sheath stained in blue. **(D)** Detail of macroscopic, globose colony stained with ACN showing the release of young, rounded colonies from the periphery of the big colony that contains cell filaments and an extensive extracellular polymeric matrix. **(E)** Praemordia with heterocytes under nitrogen-depleted conditions with small cells that are compressed. **(F)** Release of unsheathed hormogonia (white arrow) from the periphery of globose macro-colonies. Note the granulation of the cells. **(G)** Indian ink staining showing the limited sheath matrix of macro-colonies. **(H)** Globose macro-colony releasing unsheathed hormogonia to the top and smaller packets of compressed cells at the bottom. **(I)** SEM images of cut cells arranged as a filament (white arrows indicating cell–cell contact) with their thylakoid membranes (black arrows) and granular inclusions (gray arrow).

**FIGURE 8:**
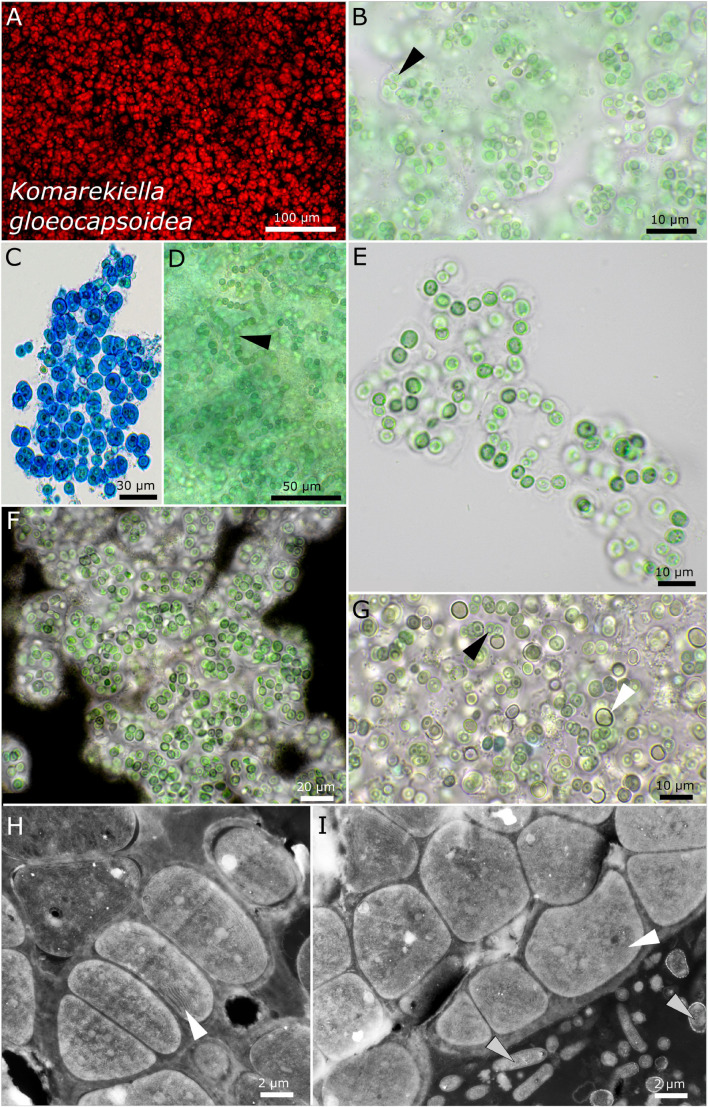
Micrographs of *Komarekiella gloeocapsoidea*. **(A)** Autofluorescence, **(B–G)** light microscopy, and **(H,I)** transmission electron microscopy (TEM) of lichen cross-sections. **(A)** Overview showing the *Gloeocapsa*-like single cell habitus of the species. **(B)** Praemordium-like stadium of the colony with wide but limited sheaths and the vacuole-like structure in the center of the cells (white arrow). **(C)** ACN stain dyeing the wide and limited sheaths of the cells of which always only a few are clustered together resembling *Gloeocapsa*. **(D)** Filamentous state only observed during heavy contamination with heterotrophic bacteria. Note the typical round, vacuole-like structure in the center of the cells (black arrow). **(E)** Rare observation of filaments. **(F)** Indian ink stain highlighting the limited and wide sheath matrix of the species. **(G)** Growths of heterocytes (white arrow) under nitrogen-depleted conditions. Note that only a few short filaments are formed, leaving most cells without contact to heterocytes. The black arrow points toward the rounded, vacuole-like structure in the center of the cells. **(H,I)** TEM images showing the photobiont cells arranged in filaments and their thylakoid membranes (white arrows) as well as the fungal hyphae of the mycobiont [bottom right in **(I)**, gray arrows].

Strains DSM 112644 and DSM 112645 form filaments with heterocytes that were mainly visible under nitrogen-depleted conditions. While strain DSM 112645 is characterized by macroscopic, up to 3-mm large, rounded colonies made of *Nostoc*-like filaments (Figure 7), DSM 112644 is formed mostly of single cells up to a few clustered cells embedded in a common sheath, resulting in a *Gloeocapsa*-like morphology. Frequently, a rounded, vacuole-like structure can be observed in the middle of the older cells. The thylakoid membranes of both species are arranged in parallel orientations in the cell periphery (Figure 7I, 8H,I).

None of the tested unicellular strains had motile cells during any of their life stages, but the filamentous strains DSM 112644 and 112645 had motile hormogonia.

In addition, the studied unicellular strains were not able to grow on BG 11 medium without nitrogen. Clear signs of chlorosis were instead visible, such as bleached cells and massive EPS production (not shown). After 4 months on nitrogen-free agar plates, the strains were transferred to BG 11 plates containing nitrogen, and all turned green in the course of a few days (not shown).

### Taxonomy

#### *Aliterella compacta* sp. nov. P. Jung, B. Büdel et M. Lakatos (Figure 2)

##### Description

Unicellular thallus is extended, compact, irregular in shape, and composed of numerous colonies or isolated ensheathed cells. The colonies on agar are blue-green, single, irregularly rounded, and of various sizes that aggregate to bigger uneven structures that become olive green to light brown when old. The cells aggregate into larger, loose clusters, with various single cells tightly encapsulated in a common sheath when young. Within these smaller packets, the young cells are oval to ellipsoid but slightly geometrically and vivid blue-green, with longitudinal diameter of 2.4–2.8 and transverse diameter of 1.4 ± 0.2 μm. It forms mostly single cells that are loosely embedded in a common sheath during aging and that are mostly rounded and only sometimes slightly ellipsoidal, 3.5–4.5 μm in diameter and pale blue-green, and surrounded by a hyaline, diffluent to sometimes limited sheath. Solitary cells without sheath were rarely observed. Cell content is mostly homogeneous, sometimes slightly finely granulated with parietal thylakoid position. The sheath is up to 5 μm wide, hyaline, sometimes lamellate, limited, and not following the shapes of the cells but rather rounded. Reproduction is by simple binary cell division in three or more planes and forms baeocytes by successive fission containing up to hundreds of cells in a common sheath; nanocytes were not observed. The cells are not motile and were not able to grow on BG 11 medium without nitrogen.

##### Habitat

Photobiont of *Peltula clavata* (Krempelhuber) Wetmore from Australia, Queensland, seepage rock 2 m above river. Leg. B. Büdel, August 1987, Nr. 18051b.

##### Etymology

*Compacta*—from Latin “compact” due to the smaller packets comprising various compressed cells.

##### Type locality

Australia.

##### Holotype

The preserved holotype specimen of the cyanobacterium is available *via* the Herbarium Hamburgense, Hamburg, Germany (HBG-025115). This was prepared from the living strain which was the source of the 16S–23S ITS rRNA gene sequence deposited at GenBank with accession number MZ160912.

##### Reference strain

The reference strain is available *via* the culture collection DSMZ Braunschweig (DSM 112641).

Phylogenetic relation and secondary structure of the 16S–23S ITS gene: Based on the 16S rRNA gene, *Aliterella compacta* shares 98.21% identity with *A. gigantea*, 97.46% with *A. chasmolithica*, 96.81% with *A. atlantica*, 96.97% with *A. antarctica*, and 98.45% with *A. shaanxiensis*. Within the D1–D1′ domain of the secondary structure, *A. compacta* has two big loops compared to all other species and two small loops formed by single-base insertions within the Box B structure (Supplementary Figure 1).

Differentiation against other species: Compared to *A. atlantica*, *A. chasmolithica*, *A. shaanxiensis*, *A. gigantea*, and *A. antarctica*, the described species *A. compacta* has smaller single cells combined in packages with a firm and limited sheath.

#### *Aliterella gigantea* sp. nov. P. Jung, B. Büdel et M. Lakatos (Figure 3)

##### Description

It is unicellular. The colonies on agar are brownish on the outside but greenish to blue-green in the inside, single, regularly rounded, and emerging up to 3 mm from the agar. It forms rounded ball-like cell clusters of blue-green color in liquid cultures and which do not float. The cells aggregate into larger, limited clusters, with various single cells tightly, but never compressed, encapsulated in a common sheath. The aggregates are rounded to spherical when young but enlarge to irregularly rounded patches during aging. The cells are oval, vivid blue-green with a longitudinal diameter of 4.8–5.3 μm and with a transverse diameter of 3.0–3.2 μm. The cells from the outer periphery of large colonies are brownish. The sheath is hyaline, firm, and limited but turns yellow to brown during aging or at the periphery of older, macroscopic colonies. The sheath capsules of single cells embedded in cell clusters are frequent; here the sheath is firm and strictly limited, forming a capsule. Solitary cells without sheath were frequently observed in case of deliberated cells. The cell content is mostly homogeneous, sometimes slightly finely granulated, with hyaline granules positioned at the outer periphery of the cells and with parietal thylakoid position. Reproduction is by simple binary cell division in three or more planes, forming baeocytes by successive fission containing up to hundreds of cells embedded in a common sheath; nanocytes were not observed. The cells are not motile and were not able to grow on BG 11 medium without nitrogen.

##### Habitat

Photobiont of *Peltula capensis* (Brusse) Büdel from the South Africa, Limpopo Province, Vhembe Nature Reserve, next to the border to Botswana and Zimbabwe, collected in 1994. The lichen grew on temporary submerged sandstone, in a seasonal flooded riverbed 600 m above sea level.

##### Etymology

*Gigantea*—from Latin “gigantic” due to the big cells compared to other known *Aliterella* species.

##### Type locality

South Africa.

##### Holotype

The preserved holotype specimen of the cyanobacterium is available *via* the Herbarium Hamburgense, Hamburg, Germany (HBG-025116). This was prepared from the living strain which was the source of the 16S–23S ITS rRNA gene sequence deposited at GenBank with accession number MZ160909.

##### Reference strain

The reference strain is available *via* the culture collection DSMZ Braunschweig (DSM 112646).

Phylogenetic relation and secondary structure of the 16S–23S ITS gene: Based on the 16S rRNA gene, *Aliterella gigantea* shares 98.21% identity with *A. compacta*, 99.17% with *A. chasmolithica*, 96.91% with *A. atlantica*, 97.56% with *A. antarctica*, and 98.26% with *A. shaanxiensis*. Within the D1–D1′ domain of the secondary structure, *A. gigantea* can be separated from all other species by a single loop formation in the stalk of the domain, a region usually marked by two loops. Within the structure of box B, a single loop that all other species have in the stalk region is missing (Supplementary Figure 1).

Differentiation against other species: Compared to *A. atlantica*, *A. chasmolithica*, *A. shaanxiensis*, *A. antarctica*, and *A. compacta*, the described species *A. gigantea* has single cells that are not compressed when combined in clusters.

#### *Sinocapsa ellipsoidea* sp. nov. P. Jung, B. Büdel et M. Lakatos (Figure 4)

##### Description

It is unicellular. Young single colonies on agar are slimy and diffluent, which combine into comprehensive, flat, and slimy biofilms, forming thick, rigid, flake-like aggregates of vivid green color during aging. The unicellular cells irregularly aggregated, but not squeezed, in dense colonies with irregular round to elongated cells that are curved when elongated, with longitudinal diameter of 4.1–4.3 μm and transverse diameter of 1.9–2.7 μm. The sheath is variable but always colorless, tight, and not lamellate; it is diffluent when forming large colonies, limited, and rigid, forming smaller round cell packages, turn capsule-like when comprising single cells or two cells and absent in single cells and freshly released or liberated cells; the sheath turns yellow in old cultures. The cell content is mostly homogeneous, sometimes slightly finely granulated to granulated when older, with parietal thylakoid position. Reproduction is by simple binary cell division in three or more planes, forming baeocytes by successive fission containing up to hundreds of cells embedded in a common sheath; nanocytes were not observed. The cells are not motile and were not able to grow on BG 11 medium without nitrogen.

##### Habitat

Photobiont of the saxicolous lichen *Peccania cerebriformis* Henssen and Büdel Austria, near Graz, on rock; Leg. A. Henssen, October 1979, Nr. 26320.

##### Etymology

*Ellipsoidea*—from Latin “elliptical,” describing the curved longitudinal shape of the cells.

##### Type locality

Austria.

##### Holotype

The preserved holotype specimen of the cyanobacterium is available *via* the Herbarium Hamburgense, Hamburg, Germany (HBG-025117). This was prepared from the living strain which was the source of the 16S–23S ITS rRNA gene sequence deposited at GenBank with accession number MZ160913.

##### Reference strain

The reference strain is available *via* the culture collection DSMZ Braunschweig (DSM 112642).

##### Phylogenetic relation and secondary structure of the 16S–23S ITS gene

*Sinocapsa ellipsoidea* and other isolates of *S. zengkensis* share 97.88–98.84% identity of the 16S rRNA gene. Within the secondary structures, the two species can be separated by a simple circular loop in the D1–D1′ domain of *S. ellipsoidea* and a more complex structure of the Box B domain (Supplementary Figure 1).

##### Differentiation against other species

The only other species *S. zengkensis* has prominent granules within the cell, and the cells are not ellipsoidal, with a slight curvature such as those of *S. ellipsoidea*. The baeocytes of *S. ellipsoidea* are irregular rounded compared to those of *S. zengkensis*.

#### *Pseudocyanosarcina* gen. nov. P. Jung, B. Büdel et M. Lakatos (Figure 5)

##### Description

This is unicellular. It forms macroscopic rounded, ball-like baeocytous colonies in liquid culture and thick three-dimensional layers on agar. The cells are large compared to other Chroococcidiopsidales, up to 8 μm; the single cells are polygonal but mostly tightly pressed against each other in rounded colonies of a few dozens of cells held together by a very firm, rigid, colorless, and limited sheaths. Reproduction by simple binary cell division in three or more planes; baeocytes or nanocytes were not observed.

##### Etymology

*Pseudocyanosarcina*—This means that it looks like Cyanosarcina, a pleurocapsalean cyanobacteria genus that shares morphological features.

#### Type Species

*Pseudocyanosarcina phycocyania* P. Jung, B. Büdel et M. Lakatos.

##### Comments

Phycocyanin granules such as those of *P. agglomerate* might be a valid differentiation criterion besides the (large) cell size.

#### *Pseudocyanosarcina phycocyania* sp. nov. P. Jung, B. Büdel et M. Lakatos (Figure 5)

##### Description

This is unicellular. It forms three-dimensional, rounded, baeocyte-like colonies of different sizes on agar when young and which aggregate into bigger clots of about 3-mm thickness. It forms dark green to blackish rounded structures of about 1–3 mm in diameter that do not float in liquid culture, without single cells swimming in the medium. The cells dyed in liquid medium (but not agar plates) were pinkish, probably due to the released phycocyanin granules of dead cells. It forms dense, rounded, bubble-like colonies that are tightly agglomerated, resembling baeocytes, comprising a few to several dozens of cells. The cells are blue-green to bright green, of irregularly rounded shape of (4.7) 5.8–6.1 (8.9) μm in diameter and tightly pressed against each other within rounded colonies, very firmly held together by a limited, slightly lamellate, not diffluent, colorless sheath. Rarely observed are single cells of polygonal to rounded, ellipsoidal shape that mostly adhere to the bigger colonies and are unsheathed. The cell content is granulated, with a few small granules and bigger phycocyanin granules of up to 3 μm that could be observed throughout a variety of tested media, light conditions, and temperatures using light microscopy (blue), simple polarized light (red), and under autofluorescence. The arrangement of the thylakoid membranes is lamellar and parietal, not filling out the whole cell. Reproduction is by simple binary cell division in three or more planes. The cells are not motile and were not able to grow on BG 11 medium without nitrogen.

##### Habitat

Photobiont of the saxicolous lichen *Peltula clavata* (Kremp.) Wetmore from Queensland, Australia, seepage rock 2 m above the river. Leg. B. Büdel, August 1987, Nr. 18051a.

##### Etymology

Phycocyanin—the name of the blue pigment unique for cyanobacteria that can be found as granules in the cells.

##### Type locality

Australia.

##### Holotype

The preserved holotype specimen of the cyanobacterium is available *via* the Herbarium Hamburgense, Hamburg, Germany (HBG-025114). This was prepared from the living strain which was the source of the 16S–23S ITS rRNA gene sequence deposited at GenBank with accession number MZ160910.

##### Reference strain

The reference strain is available *via* the culture collection DSMZ Braunschweig (DSM 112640).

Phylogenetic relation and secondary structure of the 16S–23S ITS gene: Based on the 16S rRNA gene, *Pseudosarcina phycocyania* has an identity of about 93%, with *Aliterella* species as the most related chroococcidiopsidalean species. The D1–D1′ domain of the secondary structures is marked by a complex loop structure emerging from a big loop at the stalk region, and the Box B is similar to those of the *Chroococcidiopsis* species (Supplementary Figure 1).

##### Differentiation against other species

The species can be differentiated from other chroococcidiopsidalean genera by its comparatively large cell size, phycocyanin granules, and also forming macroscopic, strictly rounded structures in liquid cultures. Although the morphology reminds on the pleurocapsalean *Cyanosarcina*, the described strain differs by a less regular arrangement of the cells that are more tightly pressed together than in *Cyanosarcina*.

#### *Compactococcus* gen. nov. P. Jung, B. Büdel et M. Lakatos (Figure 6)

##### Description

This is unicellular. The colonies tend to be structured in smaller fractions of up to a few hundred cells. Those fractions show a regular pattern of more or less linearly ranked cells with a limited, firm, and rigid sheath that follows the rectangular array of ensheathed, more or less regularly rounded, block-like cells of about 4.5 μm in diameter. The cells become bloat by vacuole-like structures during aging and aggregate crystals and fine granules in their center. Reproduction is by simple binary cell division in three or more planes. Nanocytes were not observed.

##### Etymology

*Compacto*—from Latin “compact”; *coccus*—from Latin “rounded cell,” describing the equal rectangular pattern formed by the sub-colonies.

##### Type species

*Compactococcus sarcinoides* P. Jung, B. Büdel et M. Lakatos.

#### *Compactococcus sarcinoides* sp. nov. P. Jung, B. Büdel et M. Lakatos (Figure 6)

##### Description

This is unicellular. The colonies on agar are three-dimensional, with single, rounded colonies of equal size that aggregate to bigger structures during aging, olive green to light brown. The colonies tend to be structured in smaller fractions of up to a few hundred cells. Those fractions show a regular pattern of linearly ranked cells with a limited, firm, and rigid sheath that follows the rectangular array of the cells. Single cells are more or less regularly rounded to slightly polygonal, blue-green to olive green, and (3.5) 4.5–4.8 (5.8) μm in diameter. Each single cell is always encapsulated by an up-to-4-μm-thick, sometimes lamellate, transparent, limited, and never diffluent sheath. The sheath together with the cell is pressed toward other cells in a way that it has an almost quadratic to rectangular block-like shape. Single cells stick to the package-like colonies and are always ensheathed. It never forms structures of several cells encapsulated in a common sheath and has no baeocytes. Two cells were only rarely observed to be encapsulated by a common sheath. There are coiled to tubular parietal thylakoid membranes, and cell content is not homogeneously distributed within the cells, which is increased during aging where vacuole-like structures can bloat the cell and make up to two-thirds of the cell. Fine granules together with crystals tend to concentrate in the middle of the cells during aging. Reproduction is by regular and timed binary cell division in three or more planes. Baeocytes or nanocytes were not observed, but aggregated cell clusters that are not comprised in a common sheath might be interpreted as baeocytes. The cells are not motile and were not able to grow on BG 11 medium without nitrogen.

##### Habitat

Photobiont of the saxicolous lichen *Gonohymenia* sp. Australia collected 1987. Leg. A. Henssen Nr. 27283.

##### Etymology

*Sarcinoides*—from Latin “resembling packages” due to the equal patterns formed by the ensheathed, block-like cells.

##### Type locality

Australia.

##### Holotype

The preserved holotype specimen of the cyanobacterium is available *via* the Herbarium Hamburgense, Hamburg, Germany (HBG-025112). This was prepared from the living strain which was the source of the 16S–23S ITS rRNA gene sequence deposited at GenBank with accession number MZ160914.

##### Reference strain

The reference strain is available *via* the culture collection DSMZ Braunschweig (DSM 112643).

##### Phylogenetic relation and secondary structure of the 16S–23S ITS gene

Based on the 16S rRNA gene, *Compactococcus sarcinoides* has the closest identity with nostocalean sequences (92.5%), with *Gloeocapsopsis dulcis* AAB1 being the closest related chroococcidiopsidalean species sharing 92.35% identity. The D1–D1′ region of the secondary structures of *C. sarcinoides* has a small loop at the tip and four loops within the Box B domain (Supplementary Figure 1).

##### Differentiation against other species

This species can be differentiated from other chroococcidiopsidalean genera and species by the vacuole-like structures during aging of cultures and the block-like, ensheathed form of the cells.

#### *Komarekiella globosa* sp. nov. P. Jung, B. Büdel et M. Lakatos (Figure 7)

##### Description

The colonies on agar are rounded, rigid, and firm, forming blackish to dark green rounded balls of up to 3-mm diameter in liquid culture that do not float or disintegrate into single cells. The cells are always arranged in rounded to irregularly rounded structures surrounded by a rigid, strictly limited, and thick mucilaginous, hyaline, non-lamellate sheath. Within these up to macroscopic globose structures, rounded cells of 4.7–5.1 μm in diameter form long, strictly uniseriate filaments, rarely with heterocytes. The ruptured sheaths release short, unsheathed hormogonia of similar morphology or short filaments made of only a few cells embedded in a thick, firm, non-lamellate, and hyaline sheath. The later type is often made of smaller cells that can be tightly pressed against each other. Oval heterocytes of 5.4–6.2 (6.8) μm in diameter can rarely be observed, but their number drastically increases under nitrogen-depleted conditions within smaller packages of cells (praemordia) comprising a few dozen cells. The thylakoid membrane orientation is parietal in parallel stacks.

##### Habitat

Photobiont of the lichen species *Lichinella cribellifera* (Nyl.) P. Moreno and Egea from Fuerteventura, Canary Islands, Spain, Leg. A. Henssen, August 1989. Nr. 32053c.

##### Etymology

*Globosa* from Latin, which means “globose” due to the rounded form of the colonies that reach up to 3 mm in size.

##### Type locality

Fuerteventura, Canary Islands, Spain.

##### Holotype

The preserved holotype specimen of the cyanobacterium is available via Herbarium Hamburgense, Hamburg, Germany (HBG-025113). This was prepared from the living strain which was the source of 16S–23S ITS rRNA gene sequence deposited at GenBank with accession number MZ160911.

##### Reference strain

The reference strain is available *via* the culture collection DSMZ Braunschweig (DSM 112645).

Phylogenetic relation and secondary structure of the 16S–23S ITS gene: *Komarekiella globosa* shares 98.21% identity of the 16S rRNA gene with *K. gloeocapsoides*, 98.51% with *K. atlantica*, and 97.98% with *Parakomarekiella sesnandensis*. The secondary structures differ from those of the other species by four loops in the tip region in the D1–D1′ domain and two loops of an almost equal size of the Box B region (Supplementary Figure 1).

##### Differentiation against other species

The tendency of this species to build macroscopic rounded structures in liquid medium can act as an easy trait to differentiate this species from others.

#### *Komarekiella gloeocapsoidea* sp. nov. P. Jung, B. Büdel et M. Lakatos (Figure 8)

##### Description

The colonies on agar form a dense creeping, flat, irregular mat, growing radially from the center *via* hormogonia release, forming dull blue-green, loose flocks in liquid medium. The colonies are formed by (mostly single) cells surrounded by a limited, hyaline, sometimes slightly lamellate, sheath with short uniseriate filaments. Mostly, uniseriate short filaments are formed by only a few cells that are loosely connected that stretch out to praemordia-like aggregates comprising only a few dozen cells. Heterocytes are only visible under nitrogen-depleted conditions forming apical and within short rows of cells. The heterocytes are rounded or spherical, 5.3–5.4 μm in diameter. The cells are round, 4.4–4.6 μm in diameter, dull blue-green, finely granulated, and have a characteristic vacuole-like structure centered in the cells. The thylakoid membrane orientation is parietal in parallel stacks.

##### Habitat

Photobiont of the saxicolous lichen *Gonohymenia* sp. from Czechia, Leg. A. Henssen, November 1981, Nr. 27680c.

##### Etymology

*Gloeocapsoidea*—from Latin “looks like *Gloeocapsa*” due to the morphology of the adult stage resembling *Gloeocapsa*.

##### Type locality

Czechia.

##### Holotype

The preserved holotype specimen of the cyanobacterium is available *via* the Herbarium Hamburgense, Hamburg, Germany (HBG-025111). This was prepared from the living strain which was the source of the 16S–23S ITS rRNA gene sequence deposited at GenBank with accession number MZ160908.

##### Reference strain

The reference strain is available *via* the culture collection DSMZ Braunschweig (DSM 112644).

Phylogenetic relation and secondary structure of the 16S–23S ITS gene: *Komarekiella gloeocapsoidea* shares 98.21% identity of the 16S rRNA gene with *K. globosa*, 99% with *K. atlantica*, and 97.81% with *Parakomarekiella sesnandensis*. The secondary structures differ from those of the other species by a big loop in the stalk region in the D1–D1′ domain and two small loops at the tip of the Box B region (Supplementary Figure 1).

Differentiation against other species: This species can be differentiated against other *Komarekiella* and *Parakomarekiella* species by forming colonies made of single cells or short filaments comprising only a few cells and a vacuole-like structure centered in the cells.

##### Comments

Longer filaments of the *Nostoc*-type were observed only during massive colonization of heterotrophic bacteria. Although heterocytes are formed under a nitrogen-depleted condition, growth is massively inhibited, probably due to the single-celled to short-filament nature of the species where only a few cells are connected to heterocytes.

## Discussion

### The Lichens *Peltula*, *Peccania*, *Lichinella*, and *Gonohymenia*

The order Lichinales comprises exclusively cyanobacterial lichens in the four families *Gloeoheppiaceae* (three genera), *Heppiaceae* (five genera), *Lichinaceae* (43 genera), and *Peltulaceae* (one genus) (Lücking et al., 2017). The family *Lichinaceae* comprises roughly 250 species and the *Peltulaceae* include roughly 45 species. However, little insights are given into the phylogenetic relations of the mycobionts (Schultz, 2000; Kauff et al., 2018), and mostly morphological data about their photobionts are given (Paran et al., 1971; Büdel and Henssen, 1983).

Of these, one of the best-studied genera is the saxicolous/terricolous genus *Peltula* that was found in East Africa (Swinscow and Krog, 1979), Southern Africa (Büdel, 1989; Becker, 2002), North Africa (Egea, 1989), India (Upreti and Büdel, 2018), Mexico (Büdel and Nash, 1993), and other areas such as Europe (Egea, 1989; Marques et al., 2013), the United States of America (Wetmore, 1970; Büdel and Nash, 2002), and Australia (Filson, 1988; Büdel, 2001; McCarthy, 2001). At the time when most of the scientific work about *Peltula* was conducted, the unicellular cyanobacterial genus *Chroococcidiopsis* (together with *Gloeocapsa*) was the only known unicellular terrestrial genus that could be considered as being the correct identification for the unicellular photobiont common to all species within *Peltula*. This was also supported by findings of free-living unicellular cyanobacteria that shared the morphology of the photobionts from neighboring *Peltula* lichens growing attached to the same substratum (Büdel et al., 1997; Büdel, 1999).

Besides the initial evidence that all lichen species within specific cyanolichen genera such as *Peltula*, *Peccania*, *Lichinella*, or *Gonohymenia* possess unicellular cyanobacterial photobionts that resemble the morphology of *Chroococcidiopsis* (or *Gloeocapsa*), soon it became apparent that the morphology of *Peltula* and other lichens with photobionts of the “*Chroococcidiopsis* type” showed a variation. Paran et al. (1971) already described the photobionts of *Gonohymenia mesopotamica* (syn. *Lichinella cribellifera*) and *G. sinaica* as *Gloeocapsa*-like but, at the same time, stated that cells could be found in groups and also identified ultrastructures comparable to those of nostocalean cyanobacteria. Later on, in 1983, Büdel and Henssen showed that morphology of the unicellular cyanobacterial photobiont of the “*Chroococcidiopsis* type” from the lichens *Anema nummularium*, *Peccania* sp., and *Psorotichia* sp. varied, for example, in cell size and type of baeocyte formation.

Since that time, not a single study dealt with the photobionts of these cyanolichens, although a powerful approach to taxonomically assign cyanobacteria arose in 2014 (Komárek et al., 2014). The so-called polyphasic approach as the golden standard to apply and interpret the phylogenetic position of cyanobacteria facilitates a closer look in order to untangle the black box of photobiont diversity in cyanolichens. However, as scientific work on these lichens as well as herbarium material is highly limited and the isolation of their photobionts is a sophisticated process hampered by the exceptionally slow growth, this work is restricted and could not find general patterns in the photobiont diversity. Our study showed that the photobionts of the lichen genus *Peltula* comprise specimens at least from the cyanobacterial genus *Aliterella* and the novel genus *Pseudocyanosarcina* (Figure 1A) and also from a yet to be described third genus (Figure 1C). Part of this study was also two specimens identified as *Peltula clavata* from Australia whose photobionts could be identified as *Aliterella compacta* sp. nov. and *Pseudocyanosarcina phycocyania* gen. nov. et sp. nov., indicating either a low photobiont specificity of *P. clavata*, two different *Peltula* species that can only be unveiled by phylogenetic approaches for the mycobiont, or that one of the two isolation procedures did not hit the true photobiont but rather an accompanying external cyanobacterium. In addition, the photobiont of the lichen species *P. capensis* from South Africa could be determined as *Aliterella gigantea* sp. nov., while the photobiont of *P. clavata* from the same location supports the establishment of a new genus related to *Pseudocyanosarcina* (Figure 1C). Although no clear photobiont genus could be assigned to the lichen genus *Peltula* in this study, it can be stated that no photobiont of the four investigated lichen specimens fell within the cluster of *Chroococcidiopsis sensu stricto*. It remains open if this pattern will be changed during future investigations on other *Peltula* species.

So far, the cyanolichen species *Peccania cerebriformis* was reported from the Canary Islands, Spain (Henssen et al., 1985), Teheran, Iran (Shahla et al., 2008), and the Canary Islands, Spain (Schultz and van den Boom, 2007), but besides morphological evidence (Büdel and Henssen, 1983), this is the first study to apply the polyphasic approach to photobionts from this lichen genus, assigning it to the cyanobacterial genus *Sinocapsa* as *Sinocapsa ellipsoidea* sp. nov. (Figures 1A,C).

The photobionts of the two European lichens *Gonohymenia* sp. (Czechia) and *Lichinella cribellifera* (Canary Islands, Spain) could both be assigned to the heterocytous genus *Komarekiella*, while the photobiont from the Australian specimen represented a member of the novel genus *Compactococcus* that clustered together with nostocalean strains (Figures 1A,C). Interestingly, this strain did not share morphological features with nostocalean genera but with Chroococcidiopsidales (Figure 6). It remains unanswered if a missing photobiont pattern within the lichen genus *Gonohymenia* is caused by geographic distances, photobiont selectivity in specific *Gonohymenia* species, or photobiont availability.

However, our study highlights that more attention needs to be paid to lichen members of the order Lichinales for clarification on the raised questions, a big black box that now got further enlightened.

### The Photobionts

Recently, previous studies indicated that our monophyletic picture of the cyanobacterial order Chroococcidiopsidales that got set up in 2014 (Komárek et al., 2014) is incomplete (Wang et al., 2019; Jung et al., 2021). Until now, the Chroococcidiopsidales comprised the genera *Chroococcidiopsis* with *C. thermalis* PCC 7203 as type strain, *Gloeocapsopsis* with *G. crepidinum* LEGE 06123 as type strain (Jung et al., 2021), *Aliterella* with *A. atlantica* CENA 595 as type strain, *Sinocapsa* with *S. zengkensis* CHAB 6571 as type strain as well as *Gloeocapsa* and *Chroogloeocystis*. Our study now widens this picture by the establishment of the two new chroococcidiopsidalean genera *Compactococcus* gen. nov. and *Pseudocyanosarcina* gen. nov. and even points toward additional genera that have to emerge in the future based on photobiont isolates (Figure 1C). Here it can be repeatedly shown that the order Chroococcidiopisdales is interrupted by some genera of the filamentous Oscillatoriales separating the genus *Chroococcidiopsis* from all other Chroococcidiopsidales, including the old and new genera besides *Pseudocyanosarcina* (Figure 1A). Phylogenetic calculations indicate even a well-supported relation (56% ML/61% BI) between a yet undefined genus, including the cultured, unicellular photobionts SAG 2023 and SAG 2026, and the Oscillatoriales genus *Microseira* (Figure 1A) that cannot be explained at this very moment. Even a simulated monophyly of the order Chroococcidiopsidales by excluding the 16S rRNA gene sequences of Oscillatoriales that cause the phylogenetic division (compare Figures 1A–C) results in two distinct chroococcidiopsidalean groups. Thus, a new framework for the taxonomic system around these genera in the future is unequivocal. The isolates investigated during this study are publicly available and offer a great opportunity to test this idea especially on the genome level in follow-up studies. Especially the close relationship between *Compactococcus* gen. nov. and the Nostocales is interesting, and taxonomic studies have already shown that, in some cases, phylogenetic positions between 16S rRNA gene sequences and genome data can lead to different results (Mareš, 2018). The discussion about the relation between Chroococcidiopsidales and Pleurocapsales has long been resolved, and we can support this by our findings of non-motile baeocytes, while Pleurocapsales possesses the ability to move for at least specific stadia during their development (Waterbury and Stanier, 1978).

The new species *Aliterella gigantea* sp. nov., *A. compacta* sp. nov., and *Sinocapsa ellipsoidea* sp. nov. differ morphologically from the described species based on several morphological characteristics, their phylogenetic position based on the 16S rRNA gene, and their main informative secondary structures (Supplementary Figure 1). They are the first report of species within their genera as lichen photobionts and provide a well-supported background based on morphological data and DNA sequences for further studies that will try to clarify the question after photobiont selectivity and specificity of the corresponding lichen genera and species. Most of the so far described species within these two genera are from aquatic environments, but our findings of terrestrial lichen photobionts broaden their biogeographic range as well as the ecological conditions under which members of the genera could occur.

The genus *Pseudocyanosarcina* gen. nov. was established based on a photobiont isolated from *Peltula clavata* from Australia and clustered with several highly similar 16S rRNA gene sequences from uncultured bacteria that were generated from blackish biofilms on Karst stones from the stone forest of Yunnan, China (Ren et al., 2018) as the only genetic information. Together they form a well-supported cluster indicated by 98% ML and 99% BI posterior probabilities (Figure 1A). Interestingly, among the order Chroococcidiopsidales, the genus *Pseudocyanosarcina* gen. nov. has a distinctive morphology reminiscent of the pleurocapsalean genus *Cyanosarcina* with comparably big polygonal to elongated cells that are pressed against each other without spaces between them. The type of strain of the genus *P. phycocyania* is characterized by blue phycocyanin granules in the cells (Figure 5) that were also excreted into the medium that got subsequently stained pinkish, probably due to the excretion and conversion of phycocyanin during aging. This finding could mark *P. phycocyania* as a great source for phycocyanin that has anticancer, antioxidant, and anti-inflammatory properties (Safaei et al., 2019) and can be used in living strains as highly sensitive bioindicators for heavy metals (Jusoh et al., 2017).

The genus *Compactococcus* gen. nov. was also found to be unique based on morphological characteristics, such as timed binary fission of the cells that result in a regular pattern made of cell rows with each single cell encapsulated in a wide sheath. The species *C. sarcinoides* did not form any type of baeocyte-like structure, formed vacuoles that bloated older cells, and synthesized crystals during aging, arranged in the center of the cells. Additionally, ACN staining showed that the periphery of the sheaths seems to be made of acidic polysaccharides (blue), while the composition of the sheaths differs toward the cells (pink) (Figures 6F,G). Interestingly, the sequence of the species formed a distinct cluster with the 16S rRNA gene sequences of uncultured bacteria, similar to *Pseudocyanosarcina phycocyania* sp. nov. in the phylogenetic tree (Figures 1A,C). Together with uncultured bacteria, they clustered close to the Nostocales where they occupy a unique position, strengthening the previous reports about the Nostocales being the closest relatives to the Chroococcidiopsidales (Fewer et al., 2002).

The two new species *Komarekiella globosa* sp. nov. as photobiont of the lichen *Lichinella cribellifera* from the Canary Islands, Spain, and *K. gloeocapsoidea* sp. nov. from *Gonohymenia* sp. from Czechia could both be assigned to the heterocytous Nostocales (Figures 1A,B). Compared to the phylogenetic overview given in Figure 1A, the great resolution of the nostocalean genera given in Figure 1B indicates that *Mojavia* is the closest genus to *Parakomarekiella* and *Komarekiella* previously reported (Soares et al., 2020). This was not as obvious as one might think since (i) previous studies identified the photobionts of other *Gonohymenia* species as unicellular *Gloeocapsa* (Paran et al., 1971) and (ii) because of some morphological characteristics such as aggregation of single cells that disguised their true nostocalean character. Even in culture, *K. gloeocapsoidea* sp. nov. only formed short filaments, if at all, and both species did only rarely form heterocytes on medium when nitrogen was provided. Although the morphology of both strains significantly differed, their 16S rRNA gene sequences were highly similar (98.21%). Their intermediate phylogenetic position between *Komarekiella* (*K. atlantica*; Hentschke et al., 2017) and *Parakomarekiella* (*P. sesnandensis*; Soares et al., 2020) might lead to a fusion of both genera, although *Parakomarekiella* only recently got established (Soares et al., 2020). There the authors state that there is no significant morphological difference between both genera, a circumstance that is even aggravated by our findings because *K. globosa* sp. nov. and *K. gloeocapsoidea* sp. nov. both have unique characteristics, but these do not result in distinct features based on which a split between the genera *Komarekiella* and *Parakomarekiella* could be supported.

Except the nostocalean species *Komarekiella globosa* sp. nov. and *K. gloeocapsoidea* sp. nov., all other chroococcidiopsidalean species could not grow on BG 11 medium without nitrogen, which is in line with the findings on other species such as *Gloeocapsopsis crepidinum* where *nif* genes responsible for nitrogen fixation could be detected but without growth on a nitrogen-free medium (Ramos et al., 2010). Instead the strains showed signs of chlorosis combined with massive excretion of extracellular polymeric substances as previously reported for *Chroococcidiopsis* (Billi and Caiola, 1996). Consequently, it can be assumed that all herein investigated lichen species with chroococcidiopsidalean photobionts acquired them most likely not for their nitrogen fixation abilities.

As a concluding remark, our study showed that the 16S rRNA gene phylogeny, considering ecological data and morphological characteristics, is able to reach a taxonomic resolution that is sufficient to describe and differentiate new species. So far, this has not been possible yet for lichen photobionts of the genus Nostocales (O’Brien et al., 2005). It might be necessary to add a stronger emphasis to morphological characteristics in case of members from this genus, a thesis that has to be tested in future studies.

## Conclusion

The finding of new species and even two new genera in close vicinity of the traditional genus *Chroococcidiopsis* based on isolates from cyanolichens was surprising. Instead of simplifying and clarifying the phylogenetic relationship of unicellular cyanobionts belonging to Chroococcidiopsidales, our results reveal an even more complex phylogeny and thus further open the gap. However, now we presented well-founded approaches to systematically close the open gap and not only demonstrated the value of the polyphasic approach itself but also highlight that cyanolichen photobionts can act as hidden keys for taxonomy. We aim to inspire the scientific community working on lichens to include classical isolation techniques and ecological approaches into their workflow instead of pure molecular data in order to create a common basis for future work, such as unialgal(-cyanobacterial) isolates deposited in publicly available culture collections. Moreover, morphological and ecological features have to be included because the establishment of cyanobacterial type strains solely on genome date has just recently been rejected by the International Committee on Systematics of Prokaryotes (the committee which governs the Prokaryotic Code) (Sutcliffe et al., 2020; Hugenholtz et al., 2021). Thus, combined approaches enable us to understand the symbiotic and unique relation between fungi and prokaryotic photosynthetic cyanobacteria, a relationship successfully existing for millions of years.

## Data Availability Statement

The datasets presented in this study can be found in online repositories. The names of the repository/repositories and accession number(s) can be found in the article/Supplementary Material.

## Author Contributions

PJ designed the study, conducted lab work, analyzed the results, and wrote the manuscript supervised by ML and BB. MS and BB contributed to the images of the lichens and details on cyanolichen taxonomy, while AD and KB conducted lab work and helped to wrote the manuscript. All authors edited the manuscript accordingly.

## Conflict of Interest

The authors declare that the research was conducted in the absence of any commercial or financial relationships that could be construed as a potential conflict of interest.

## Publisher’s Note

All claims expressed in this article are solely those of the authors and do not necessarily represent those of their affiliated organizations, or those of the publisher, the editors and the reviewers. Any product that may be evaluated in this article, or claim that may be made by its manufacturer, is not guaranteed or endorsed by the publisher.
