# Supplementary material for: Opening the Gap: Rare Lichens With Rare Cyanobionts – Unexpected Cyanobiont Diversity in Cyanobacterial Lichens of the Order Lichinales
Source: Front Microbiol. 2021 Oct 6;12:728378. doi: 10.3389/fmicb.2021.728378 (PMC8527099; doi:10.3389/fmicb.2021.728378)
Supplement: Supplementary file 1 [file Data_Sheet_1.PDF]

Supplementary Material

1 Supplementary Data

1.1 Supplementary Figures

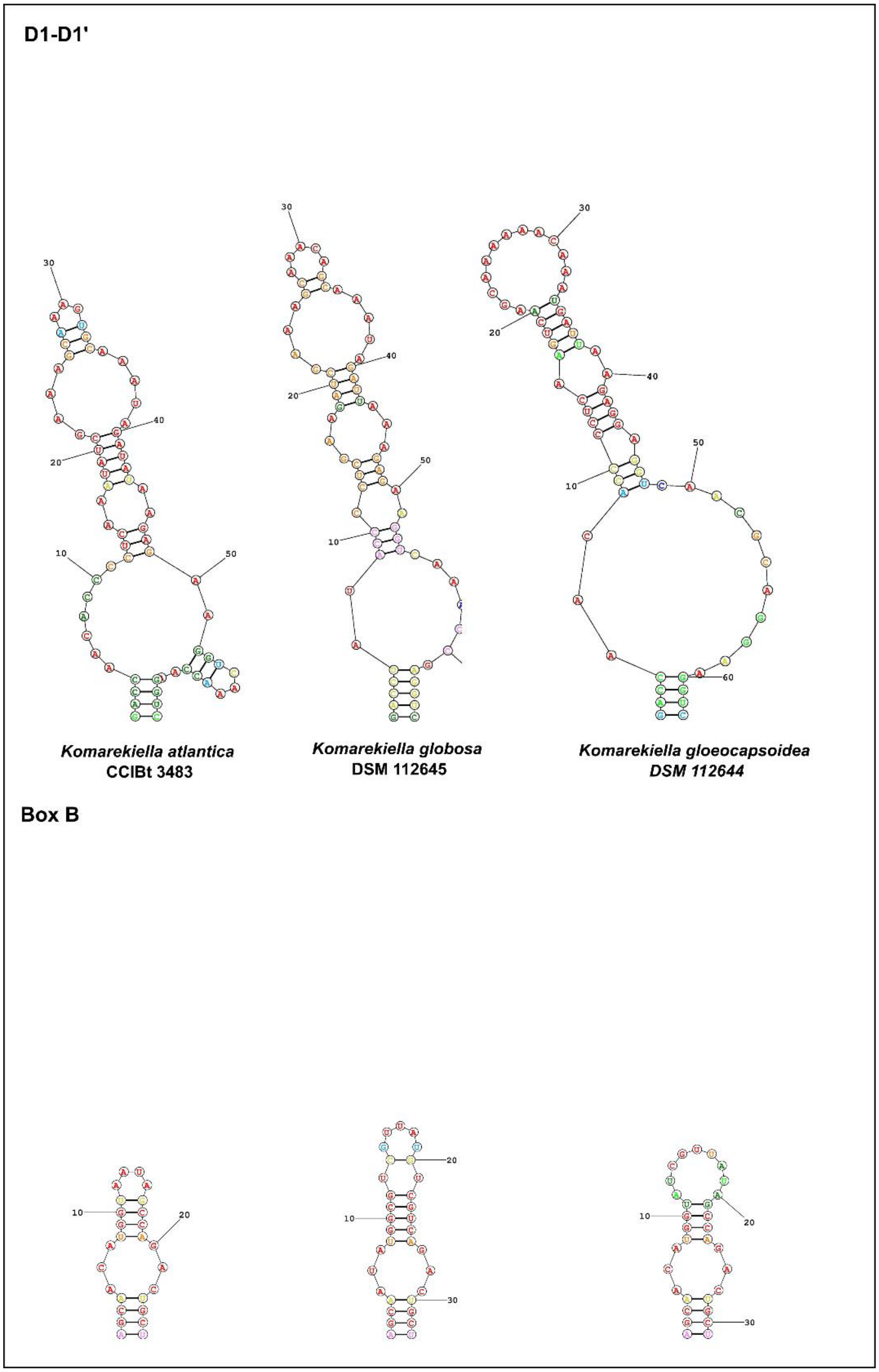

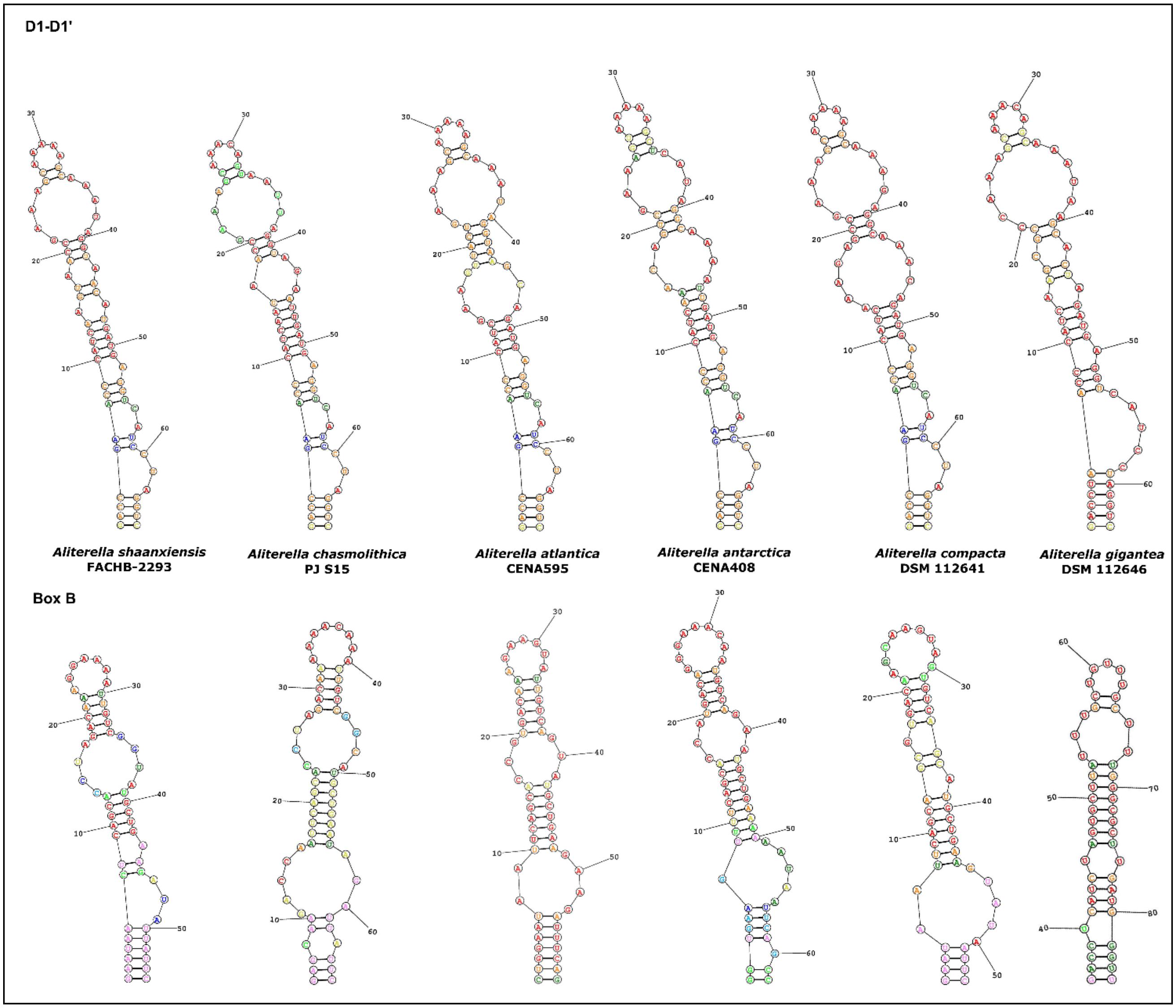
D1-D1'
Aliterella shaanxiensis FACHB-2293
Aliterella chasmolithica PJ S15
Aliterella atlantica CENA595
Aliterella antarctica CENA408
Aliterella compacta DSM 112641
Aliterella gigantea DSM 112646
Box B

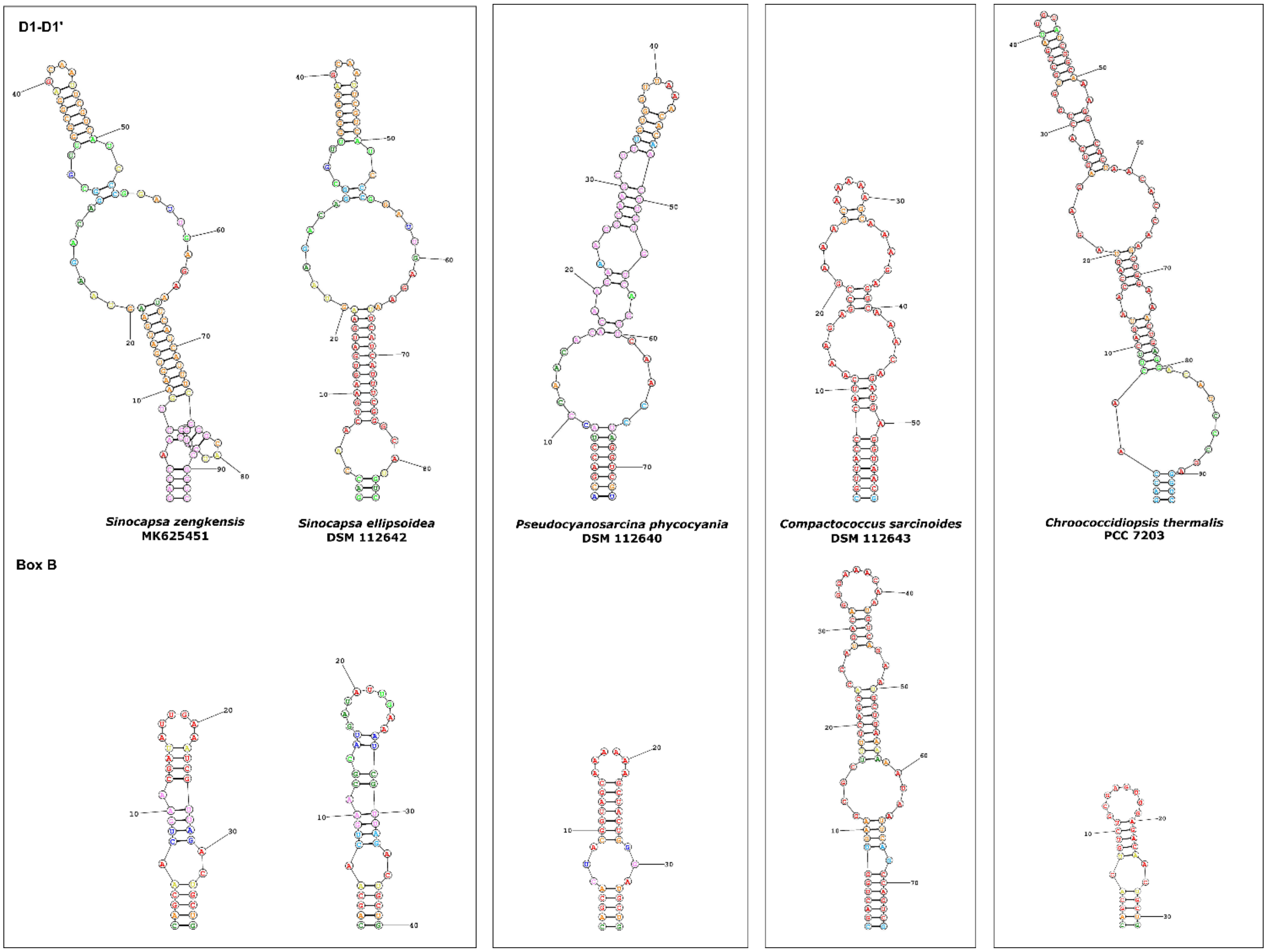

Supplementary Figure 1. Secondary structure of the main informative helices of the 16-23S ITS region of the inspected species in comparison to those of related species of the same or related genera.
